# ChemoPROphyLaxIs with hydroxychloroquine For covId-19 infeCtious disease (PROLIFIC) to prevent covid-19 infection in frontline healthcare workers: A structured summary of a study protocol for a randomised controlled trial

**DOI:** 10.1186/s13063-020-04543-4

**Published:** 2020-07-02

**Authors:** Carmel M. McEniery, Marie Fisk, Karen Miles, Fotini Kaloyirou, Annette Hubsch, Jane Smith, Ian B. Wilkinson, Joseph Cheriyan

**Affiliations:** 1 Division of Experimental Medicine & Immunotherapeutics, University of Cambridge, Cambridge, UK; 2 Cambridge University Hospitals NHS Foundation Trust, Cambridge, UK; 3 Cambridge Clinical Trials Unit, University of Cambridge, Cambridge, UK

**Keywords:** COVID-19, Randomised controlled trial, Protocol, Chemoprophylaxis, Healthcare workers, Hydroxychloroquine, Daily-dosing, Weekly-dosing, Placebo

## Abstract

**Objectives:**

**Primary objective:**

To determine whether chemoprophylaxis with hydroxychloroquine versus placebo increases time to contracting coronavirus disease 2019 (COVID-19) in frontline healthcare workers.

**Secondary objectives:**

1. To determine whether chemoprophylaxis with daily versus weekly dosing of hydroxychloroquine increases time to contracting COVID-19 disease in frontline healthcare workers.
2. To compare the number of COVID-19 cases between each trial arm on the basis of positive tests (as per current clinical testing methods and/or serology)
3. To compare the percentage of COVID-19 positive individuals with current testing methods versus serologically-proven COVID-19 in each trial arm
4. To compare COVID-19 disease severity in each trial arm
5. To compare recovery time from COVID-19 infection in each trial arm

**Exploratory objectives:**

1. To determine compliance (as measured by trough pharmacokinetic hydroxychloroquine levels) on COVID-19 positive tests
2. To determine if genetic factors determine susceptibility to COVID-19 disease or response to treatment
3. To determine if blood group determines susceptibility to COVID-19 disease
4. To compare serum biomarkers of COVID-19 disease in each arm

**Trial design:**

Double-blind, multi-centre, 2-arm (3:3:2 ratio) randomised placebo-controlled trial

**Participants:**

National Health Service (NHS) workers who have direct patient contact delivering care to patients with COVID-19.

Participants in the trial will be recruited from a number of NHS hospitals directly caring for patients with COVID-19.

**Inclusion criteria:**

To be included in the trial the participant MUST:

1. Have given written informed consent to participate
2. Be aged 18 years to 70 years
3. Not previously have been diagnosed with COVID-19
4. Work in a high-risk secondary or tertiary healthcare setting (hospitals accepting COVID-19 patients) with direct patient-facing care

**Exclusion criteria:**

The presence of any of the following will mean participants are ineligible:

1. Known COVID-19 positive test at baseline (if available)
2. Symptomatic for possible COVID-19 at baseline
3. Known hypersensitivity reaction to hydroxychloroquine, chloroquine or 4-aminoquinolines
4. Known retinal disease
5. Known porphyria
6. Known chronic kidney disease (CKD; eGFR<30ml/min)
7. Known epilepsy
8. Known heart failure or conduction problems
9. Known significant liver disease (Gilbert’s syndrome is permitted)
10. Known glucose-6-phosphate dehydrogenase (G6PD) deficiency
11. Currently taking any of the following contraindicated medications: Digoxin, Chloroquine, Halofantrine, Amiodarone, Moxifloxacin, Cyclosporin, Mefloquine, Praziquantel, Ciprofloxacin, Clarithromycin, Prochlorperazine, Fluconazole
12. Currently taking hydroxychloroquine or having a clinical indication for taking hydroxychloroquine
13. Currently breastfeeding
14. Unable to be followed-up during the trial
15. Current or future involvement in the active treatment phase of other interventional research studies (excluding observational/non-interventional studies) before study follow-up visit
16. Not able to use or have access to a modern phone device/web-based technology
17. Any other clinical reason which may preclude entry in the opinion of the investigator

**Intervention and comparator:**

Interventions being evaluated are:

A) Daily hydroxychloroquine or
B) Weekly hydroxychloroquine or
C) Placebo

The maximum treatment period is approximately 13 weeks per participant.

Hydroxychloroquine-identical matched placebo tablets will ensure that all participants are taking the same number and dosing regimen of tablets across the three trial arms. There is no variation in the dose of hydroxychloroquine by weight.

The dosing regimen for the three arms of the study (A, B, C) are described in further detail below.

**Arm A:** Active Hydroxychloroquine (– daily dosing and placebo-matched hydroxychloroquine - weekly dosing). Form: Tablets

Route: Oral. Dose and Frequency:

Active hydroxychloroquine:

- Days 1-2: Loading phase - 400mg (2 x 200mg tablets) taken twice a day for 2 days
- Days 3 onwards: Maintenance Phase - 200mg (1 x 200mg tablet) taken once daily, every day for 90 days (~3 months)

Matched Placebo hydroxychloroquine:

- Days 3 onwards: Maintenance Phase - 2 tablets taken once a week on the same day each week (every 7^th^ day) for 90 days (~3 months)

**Arm B:** Active Hydroxychloroquine (- weekly dosing and placebo matched hydroxychloroquine – daily dosing.) Form: Tablets

Route: Oral. Dose and Frequency:

Active hydroxychloroquine:

- Days 1-2: Loading Phase - 400mg (2 x 200mg tablets) taken twice daily for 2 days
- Days 3 onwards: Maintenance Phase - 400mg (2 x 200mg tablets) taken once a week on the same day each week (every 7^th^ day) for 90 days (~3 months)

Matched Placebo hydroxychloroquine:

- Days 3 onwards: Maintenance Phase - 1 tablet taken once daily for 90 days (~3 months)

**Arm C:** Matched placebo Hydroxychloroquine (- daily dosing and matched placebo hydroxychloroquine - weekly dosing.) Form: Table. Route: Oral. Frequency:

Matched placebo hydroxychloroquine - daily dosing:

- Days 1-2: Loading Phase - 2 tablets taken twice daily for 2 days
- Days 3 onwards: Maintenance Phase - 1 tablet taken once daily for 90 days (~3 months)

Matched placebo hydroxychloroquine – weekly dosing:

- Days 3 onwards: Maintenance Phase - 2 tablets taken once a week on the same day each week (every 7th day) for 90 days (~3 months)

A schematic of the dosing schedule can be found in the full study protocol (Additional File 1).

**Main outcomes:**

Time to diagnosis of positive COVID-19 disease (defined by record of date of symptoms onset and confirmed by laboratory test)

**Randomisation:**

Participants will be randomised to either hydroxychloroquine dosed daily with weekly placebo, HCQ dosed weekly with daily placebo, or placebo dosed daily and weekly.

Randomisation will be in a 3:3:2 ratio [hydroxychloroquine-(daily), hydroxychloroquine-(weekly), placebo], using stratified block randomisation. Random block sizes will be used, and stratification will be by study site.

**Blinding (masking):**

Participants and trial investigators consenting participants, delivering trial assessments and procedures will be blinded to intervention.

**Numbers to be randomised (sample size):**

A sufficient number of participants will be enrolled so that approximately 1000 participants in total will have data suitable for the primary statistical analysis. It is anticipated that approximately 1,200 participants will need to be enrolled in total, to allow for a 20% dropout over the period of the trial. This would result in approximately 450:450:300 participants randomised to hydroxychloroquine daily, hydroxychloroquine weekly+daily matched placebo or matched-placebo daily and weekly.

**Trial Status:**

V 1.0, 7^th^ April 2020

EU Clinical Trials Register

EudraCT Number: 2020-001331-26

Date of registration: 14^th^ April 2020

Trial registered before first participant enrolment. Trial site is Cambridge University Hospitals NHS Foundation Trust. Recruitment started on 11^th^ May 2020. It is anticipated that the trial will run for 12 months. The recruitment end date cannot yet be accurately predicted.

**Full protocol:**

The full protocol is attached as an additional file, accessible from the Trials website (Additional file 1). In the interest of expediting dissemination of this material, the familiar formatting has been eliminated; this Letter serves as a summary of the key elements of the full protocol.

The study protocol has been reported in accordance with the Standard Protocol Items: Recommendations for Clinical Interventional Trials (SPIRIT) guidelines (Additional file 2).

## Supplementary information

**Additional file 1.**

**Additional file 2.**

## Data Availability

Not applicable.

