# Correction to: ChemoPROphyLaxIs with hydroxychloroquine For covId-19 infeCtious disease (PROLIFIC) to prevent covid-19 infection in frontline healthcare workers: A structured summary of a study protocol for a randomised controlled trial

**DOI:** 10.1186/s13063-020-04578-7

**Published:** 2020-07-14

**Authors:** Carmel M. McEniery, Marie Fisk, Karen Miles, Fotini Kaloyirou, Annette Hubsch, Jane Smith, Ian B. Wilkinson, Joseph Cheriyan

**Affiliations:** 1 Division of Experimental Medicine & Immunotherapeutics, University of Cambridge, Cambridge, UK; 2 Cambridge University Hospitals NHS, Foundation Trust, Cambridge, UK; 3 Cambridge Clinical Trials Unit, University of Cambridge, Cambridge, UK

**Correction to: Trials 21:604 (2020)**

**https://doi.org/10.1186/s13063-020-04543-4**

Following acceptance of the original article [1], the authors notified us that Evangelia Vamvaka should be removed from the author list.
