# Supplementary material for: ChemoPROphyLaxIs with hydroxychloroquine For covId-19 infeCtious disease (PROLIFIC) to prevent covid-19 infection in frontline healthcare workers: A structured summary of a study protocol for a randomised controlled trial
Source: Trials. 2020 Jul 2;21:604. doi: 10.1186/s13063-020-04543-4 (PMC7330261; doi:10.1186/s13063-020-04543-4)
Supplement: Supplementary file 1 — Additional file 1. [file 13063_2020_4543_MOESM1_ESM.pdf]

ChemoPROphyLaxIs For covId-19 infeCtious disease (the PROLIFIC trial)

Trial Title: Chemoprophylaxis For COVID-19 Infectious Disease

Protocol Number: PROLIFIC2020

EudraCT Number: 2020-001331-26

Investigational Products: Hydroxychloroquine

Protocol Version: 1.0

Chief Investigator: Dr Joseph Cheriyan

CI Address: Clinical Pharmacology, Directorate of Acute Medical Services, Division C,
Cambridge University Hospitals NHS Foundation Trust
ACCI, Level 3, Box 98
Addenbrooke's Hospital
Hills Road
Cambridge
CB2 0QQ

Telephone: 01223 256653

Trial Sponsor: Cambridge University Hospitals NHS Foundation Trust

SAR Reporting: PROLIFIC Trial Co-ordinator

Email: cambs.cardiovascular@nhs.net

1 Protocol Signatures

I give my approval for the attached protocol entitled 'Chemoprophylaxis for COVID-19 infectious Disease (the PROLIFIC trial)' dated 7th April 2020.

Chief Investigator

Name:

Signature: ____

Date: ____________________________

Site Signatures

I have read the attached protocol entitled "Chemoprophylaxis for COVID-19 infectious Disease (the PROLIFIC trial)" dated 7th April 2020 and agree to abide by all provisions set forth therein.

I agree to comply with the conditions and principles of Good Clinical Practice as outlined in the European Clinical Trials Directives 2001/20/EC and the GCP Directive 2005/28/EC.

I agree to ensure that the confidential information contained in this document will not be used for any other purpose other than the evaluation or conduct of the clinical investigation without the prior written consent of the Sponsor

Principal Investigator

Name:

Signature: ____

Date: ____________________________

2 Protocol Contributors

**Investigators:**	
**Dr Joseph Cheriyan**	Consultant in Clinical Pharmacology, Directorate of Acute Medical Services, Division C, Cambridge University Hospitals NHS Foundation Trust & Division of Experimental Medicine & Immunotherapeutics & Cambridge Clinical Trials Unit, University of Cambridge
**Professor Ian Wilkinson**	Head of Division, Division of Experimental Medicine & Immunotherapeutics, University of Cambridge & Director, Cambridge Clinical Trials Unit, Cambridge University Hospitals NHS Foundation Trust
**Dr Carmel McEniery**	Senior Research Associate, Division of Experimental Medicine & Immunotherapeutics, University of Cambridge
**Dr Edward Banham-Hall**	Consultant in Acute Medicine, Directorate of Acute Medical Services, Division C, Cambridge University Hospitals NHS Foundation Trust
**Dr Michalis Kostapanos**	Consultant in Acute Medicine, Directorate of Acute Medical Services, Division C, Cambridge University Hospitals NHS Foundation Trust
**Dr Frances Hall**	Consultant Rheumatologist, Rheumatology Research Unit, Cambridge University Hospitals NHS Foundation Trust
**Dr Marie Fisk**	Academic Clinical Lecturer, Division of Experimental Medicine & Immunotherapeutics (EMIT), University of Cambridge & Department of Respiratory Medicine, Cambridge University Hospitals NHS Foundation Trust
**Dr Alain Vuylsteke**	Consultant in Intensive Care, Royal Papworth Hospital
**Dr Thomas Hiemstra**	Consultant Nephrologist, Experimental Medicine & Immunotherapeutics Division, Department of Medicine, University of Cambridge
**Dr Fraz Mir**	Consultant in Clinical Pharmacology/Acute Medicine, Directorate of Acute Medical Services, Division C, Cambridge University Hospitals NHS Foundation Trust
**Professor Paul Lehner**	Department of Medicine and the Cambridge Institute for Medical Research, University of Cambridge
**Dr Hoi Ping Mok**	Consultant (Locum) in Acute Medicine & Infectious Diseases, Department of Medicine, University of Cambridge
**Professor Ravindra Gupta**	Cambridge Institute of Therapeutic Immunology & Infectious Disease, University of Cambridge
**Dr Jonathan Fuld**	Consultant in Acute and Respiratory medicine, Cambridge University Hospitals NHS Foundation Trust
**Dr Ravi Mahadeva**	Consultant in Respiratory Medicine, Cambridge University Hospitals NHS Foundation Trust
**Dr Effrossyni Gkrania-Klotsas**	Consultant in Infectious Diseases, MRC Epidemiology Unit, Department of Medicine, University of Cambridge

**Annette Hubsch**	Research Nurse, Vascular Research Clinic, Cambridge University Hospitals NHS Foundation Trust
**Fotini Kaloyirou**	Research Nurse, Vascular Research Clinic, Cambridge University Hospitals NHS Foundation Trust
**Evangelia Vamvaka**	Research Nurse, Vascular Research Clinic, Cambridge University Hospitals NHS Foundation Trust
**Karen Miles**	Research Nurse, Vascular Research Clinic, Cambridge University Hospitals NHS Foundation Trust
**Jane Smith**	Research Nurse, Vascular Research Clinic, Cambridge University Hospitals NHS Foundation Trust
**Anita Chhabra**	Lead Clinical Trials Pharmacist, Cambridge University Hospitals NHS Foundation Trust

**Trial Statistician:**
**Dr Simon Bond**<br>Cambridge Clinical Trials Unit, Box 401, Cambridge University Hospitals NHS Foundation Trust, Hills Road, Cambridge, CB2 0QQ
**Trial Coordination:**
**Dr Ella James**<br>Coton House, Level 4, Box 401, Hills Road, Cambridge, CB2 0QQ<br>Telephone: 01223 349762 / 01223 250874<br>e-mail: ella.james@addenbrookes.nhs.net<br>cambs.cardiovascular@nhs.net
**Sponsor:**
**Cambridge University Hospitals NHS Foundation Trust**<br>Research &Development Department (Box 277)<br>Addenbrooke's Hospital, Hills Road<br>Cambridge, CB2 0QQ<br>Telephone: 01223 245151 e-mail:<br>ccturegulatory@addenbrookes.nhs.uk

Amendment History

Version No.	History	Date
1.0	Initial version	7th April 2020

1 Protocol Signatures **2**
2 Protocol Contributors **3**
3 Abbreviations **7**
4 Introduction **8**
5 Rationale for Trial **9**
5.1 Hypotheses **9**
6 Trial Design **9**
6.1 Statement of design **9**
6.2 Number of Participants **9**
6.3 Participant Trial duration **9**
6.4 Trial objectives **10**
6.5 Trial Outcome Measures **10**
6.6 Trial Flow Chart **11**
7 Selection and withdrawal of participants **11**
7.1 Inclusion Criteria **11**
7.2 Exclusion Criteria **11**
7.3 Treatment Assignment and Randomisation **12**
7.4 Withdrawal criteria **12**
7.4.1 Treatment Withdrawal 12
7.4.2 Participant Withdrawal 12
8 Trial Treatments **13**
8.1 Treatment summary **13**
8.1.1 Name and description of IMP 13
8.1.2 Legal Status of IMPs 13
8.1.3 Supply 13
8.1.4 Packaging and Labelling 13
8.1.5 Storage Conditions 13
8.1.6 Dispensing 14
8.1.7 Treatment duration 14
8.1.8 Dose and Administration of IMPs 14
8.1.9 Administration 15
8.1.10 Missed or replacement doses: 15
8.1.11 Contraindications 15
8.1.12 Dosage modifications 15
8.1.13 Permitted and prohibited medications 15
8.1.14 Drug interactions 16
8.1.15 Method of unblinding 16
9 Schedule of assessments **16**
9.1 Trial assessments (hospital) **16**
9.1.1 Visit 1 - Baseline 16
9.1.2 Visit 2 – Interim visit 17
9.1.3 Visit 3 17
9.1.4 Weekly (remote) Assessments 17
9.1.5 Ad hoc Assessments (triggered) 17
9.2 End of Trial Participation **18**
9.3 Trial restrictions **18**
9.4 Procedures and assessments **18**

9.5 Storage of samples 18
10 Assessment of Safety 18
10.1 Definitions 18
10.2 Safety monitoring and reporting in the PROLIFIC trial 19
10.3 Reference Safety Information (RSI) 19
10.4 Expected Adverse Reactions/Serious Adverse Reactions (AR /SARs) 19
10.5 Expected Adverse Events/Serious Adverse Events (AE/SAE) 19
10.6 Recording and Evaluating SAEs, Reporting SARs 19
10.6.1 Assessment of seriousness 20
10.6.2 Assessment of causality 20
10.6.3 Clinical assessment of severity 20
10.7 Reporting of Suspected Unexpected Serious Adverse Reactions (SUSARs) 21
10.7.1 Who should report and whom to report to? 21
10.7.2 When to report SUSARs? 21
10.7.3 How to report SUSARs? 21
10.8 Pregnancy Reporting 22
11 Statistics 22
11.1 Statistical methods 22
11.2 Number of Participants to be enrolled 22
11.3 Interim analysis 22
11.4 Criteria for the premature termination of the trial 23
11.5 Definition of the end of the trial 23
12 Data handling and record keeping 23
12.1 Source Data 23
12.2 Data Protection & Participant Confidentiality 24
13 Data Monitoring Committee 24
14 Ethical & Regulatory considerations 24
14.1 Consent 24
14.2 Ethical committee review 24
14.3 Regulatory Compliance 24
14.4 Protocol Amendments 25
14.5 Peer Review 25
14.6 Declaration of Helsinki and Good Clinical Practice 25
14.7 GCP Training 25
15 Sponsorship, Financial and Insurance 25
16 Monitoring, Audit & Inspection 25
17 Protocol Compliance and Breaches of GCP 25
18 Publications policy 26
19 References 26
20 Appendices 27
20.1 Table 1 Schedule of assessments and procedures 27
20.2 Pharmacokinetic simulations to support Hydroxychloroquine dosing 28

3 Abbreviations

AE	Adverse Event
CA	Competent Authority
Con med	Concomitant Medication
COVID-19	Coronavirus Disease 2019
CKD	Chronic Kidney Disease
CRF	Case Report Form (when used for data collection)
CRF	Clinical Research Facility
DMC	Data Management Committee
DSUR	Development Safety Update Report
ED	Emergency department
eGFR	Estimated Glomerular Filtration Rate
GP	General Practitioner
GCP	Good Clinical Practice
HCW	Healthcare workers
HCQ	Hydroxychloroquine
HDU	High dependency unit
IMP	Investigational Medicinal Product
ITU	Intensive Treatment Unit
MHRA	Medicines and Healthcare products Regulatory Agency
POC	Point of Care
PCR	Polymerase chain reaction
PK	pharmacokinetic
R&D	Research and Development
RA	Regulatory Agency
RCT	Randomised Control Trial
REC	Research Ethics Committee
SAE	Serious Adverse Event
SD	Standard Deviation
SUSAR	Suspected Unexpected Serious Adverse Reaction
TMG	Trial Management Group
TPM	Trial Procedure Manual
TSC	Trial Steering Committee

4 Introduction

The current COVID-19 infectious disease (COVID-19) pandemic, with over 800,000 confirmed cases in over 190 countries, and over 39,000 confirmed deaths (World Health Organization, 2020) is on track to become one of the largest pandemics in human history. The number of confirmed cases in the UK is rising substantially and rapidly, with the potential to overwhelm the ability of the entire National Health Service (NHS) to cope with the increased demand. Mature health systems like the NHS are also experiencing shortages in the availability of personal protective equipment (PPE), resulting in an increased risk for healthcare worker-associated infection, especially amongst those involved in direct patient care. Recent reports have highlighted that this risk could range from 20% in Bergamo[1] to 38% in Wuhan[2]. Therefore, efforts to protect these workers from development of COVID-19, using chemoprophylactic agents, require urgent evaluation.

COVID-19 was first recognised in Wuhan, Hubei Province, China in December 2019[3]. The infectious agent causing COVID-19 is SARS-CoV-2 – a coronavirus which shares some homology to the coronavirus which caused the SARS outbreak in 2003. In humans, COVID-19 is primarily a respiratory disease, and can vary from asymptomatic infection through coryzal upper respiratory tract infection to severe respiratory distress and death. Whilst the vast proportion of illness is mild within the community at large, the pathophysiology of COVID-19, in its more severe form, primarily involves the development of an acute respiratory distress syndrome (ARDS) characterized by diffuse alveolar damage, with emerging evidence suggesting that some patients respond to SARS-CoV-2 infection with a cytokine storm, resembling bacterial sepsis. Patients move through various stages of illness, including (i) a replicative stage, where the virus replicates over a period of several days and is not contained by the host’s innate immune response; (ii) an adaptive immunity stage, where an adaptive immune response is triggered, but which may also lead to increased levels of inflammatory cytokines and ensuing lung tissue damage, causing clinical deterioration. Currently there is no vaccine against SARS-CoV-2 infection and there are no specific drugs which are proven to prevent, treat or delay the progression of COVID-19 in at-risk groups. In this context, repurposing of old drugs for use as antiviral therapies may be a useful strategy for prevention and therapy, because knowledge of safety profiles, side effects and drug interactions are well understood[4].

Hydroxychloroquine (HCQ) is a widely used anti-malarial drug with immunomodulatory effects. It is an analogue of chloroquine, another anti-malarial drug, which was found to inhibit the growth of SARS-CoV-2 *in vitro*[5]. Compared with chloroquine, HCQ has fewer concerns regarding drug-drug interactions and toxicity and was also reported to have anti-SARS-CoV activity *in vitro* in a previous SARS outbreak[6]. Importantly, in a recent *in vitro* study of SARS-CoV-2 infected Vero cells[7], HCQ exhibited a superior anti-viral effect to chloroquine when administered *prior to* viral challenge with SARS-CoV-2 (EC_{50} values for chloroquine (>100 and 18.01uM) compared to HCQ (6.25 and 5.85uM) at 24 and 48 hours respectively. Moreover, the inhibitory effect of chloroquine was poor, and did not exceed 50%, even at the highest concentration of chloroquine used (Figure 1)[7].

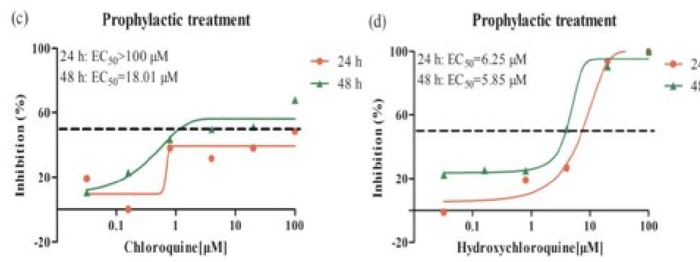

The molecular mechanism of action of HCQ has not been fully elucidated. However, previous data suggest that inhibition of SARS-CoV-2 may occur via prevention of acidification of the endosomal compartment; this reduces/prevents fusion of the SARS-CoV-2 envelope with the endosomal membrane; or inhibition of glycosylation of viral proteins and other processes, resulting in an anti-viral effect of the drug[8]. As such, HCQ

may be an effective anti-viral strategy which, if deployed early, may have created a milieu in which the virus is unable to replicate effectively and cause progression of COVID-19. As yet, there are no *in vivo* data in humans describing the chemoprophylactic effects of HCQ against SARS-CoV-2 and development of COVID-19 or the optimal dosing regimen of HCQ, although preliminary PK modelling data are available (refer to Appendix, section 20.2).

COVID-19 is an international emergency. There is now an urgent need to determine if chemoprophylaxis for healthcare workers who are exposed to a life-threatening infection due to their close proximity to COVID-19 patients will be effective. Hydroxychloroquine is well-tolerated, commonly used and its pharmacokinetic profile is well understood, making it an ideal drug to re-purpose and trial as an antiviral agent in healthcare workers who are likely to be exposed to SARs-CoV-2 in healthcare settings. If successful, this will not only sustain the limited number of such skilled healthcare professionals to continue to work in these specialised areas, but also to reduce the burden on an already strained healthcare system.

5 Rationale for Trial

The rationale for the PROLIFIC clinical trial is to reduce the risk of SARS-CoV-2 infection/COVID-19 disease in frontline healthcare workers who potentially will be exposed to the SARs-CoV-2 coronavirus.

5.1 Hypotheses

Primary: Time to COVID-19 disease will be increased significantly in trial participants randomised to HCQ compared to placebo.

Secondary: Daily dosing of HCQ further increases the time to COVID-19 disease compared to weekly dosing.

6 Trial Design

6.1 Statement of design

This trial will be a double-blind, randomised placebo-controlled trial in a cohort of frontline healthcare workers, who will potentially be exposed to SARS-CoV-2. Eligible participants will be defined as frontline NHS workers who have direct clinical contact (patient care) with COVID-19 patients, who respond to study advertisements placed in clinical areas, web-based (online/generic Trust emails/newsletters) and social media platforms.

Study sites will principally include Cambridge University Hospitals NHS Foundation Trust, Royal Papworth Hospital NHS Foundation Trust as well as other UK secondary/tertiary care centres.

6.2 Number of Participants

A sufficient number of participants will be enrolled so that approximately 1000 participants in total will have data suitable for the primary statistical analysis. It is anticipated that approximately 1,200 will be enrolled in total to allow for a 20% dropout over the period of the trial.

6.3 Participant Trial duration

The maximum duration of treatment will be approximately 13 weeks per participant.

6.4 Trial objectives

6.4.1 Primary objective

- To determine whether chemoprophylaxis with HCQ versus placebo increases time to COVID-19 disease in frontline healthcare workers.

6.4.2 Secondary objectives

- To determine whether chemoprophylaxis with daily versus weekly dosing of HCQ increases time to COVID-19 disease in frontline healthcare workers
- To compare the number of confirmed COVID-19 cases between each arm on the basis of positive tests (as per current clinical testing methods and/or serology)
- To compare the percentage of COVID-19 positive individuals with current testing methods versus serologically-proven COVID-19 disease in each arm
- To compare disease severity in each arm
- To compare recovery time in each arm

6.4.3 Exploratory objectives

- To determine compliance (as measured by trough pharmacokinetic HCQ levels) on COVID-19 positive tests
- To determine if genetic factors determine susceptibility to COVID-19 disease or response to treatment
- To determine if blood group determines susceptibility to COVID-19 disease
- To compare serum biomarkers of COVID-19 disease in each arm

6.5 Trial Outcome Measures

6.5.1 Primary outcome measure

- Time to positive COVID-19 disease

6.5.2 Secondary outcome measure

- Number of COVID-19 test positive cases
- Number of COVID-19 serological test positive cases
- Severity of COVID-19 disease between each arm$^{\delta}$ – (i) not requiring hospitalisation, requiring admission, HDU/ICU admission, death; (ii) length of personal sickness, length of inpatient stay [self-isolation due to contacts with positive household members will not be included] (iii) patient reported outcome measures$^{\diamond}$
- Incidence of common COVID-19 complications between arms (including ARDS, viral pneumonitis, myocarditis/myocardial injury, AKI)$^{\delta}$

δ= based on medical records and participant feedback where appropriate

◇ = measures are taken weekly using telephone or online app/web-based interface for duration of participant is on the trial

6.5.3 Exploratory outcome measures

- Prevalence of COVID-19 disease based on compliance data (trough pharmacokinetic data) *
- Prevalence of COVID-19 disease on the basis of underlying genetic status
- Prevalence of COVID-19 disease according to blood group
- Serum store for exploratory analysis at various scheduled time points*

*= Participants may continue with the trial if this measure cannot be taken.

6.6 Trial Flow Chart

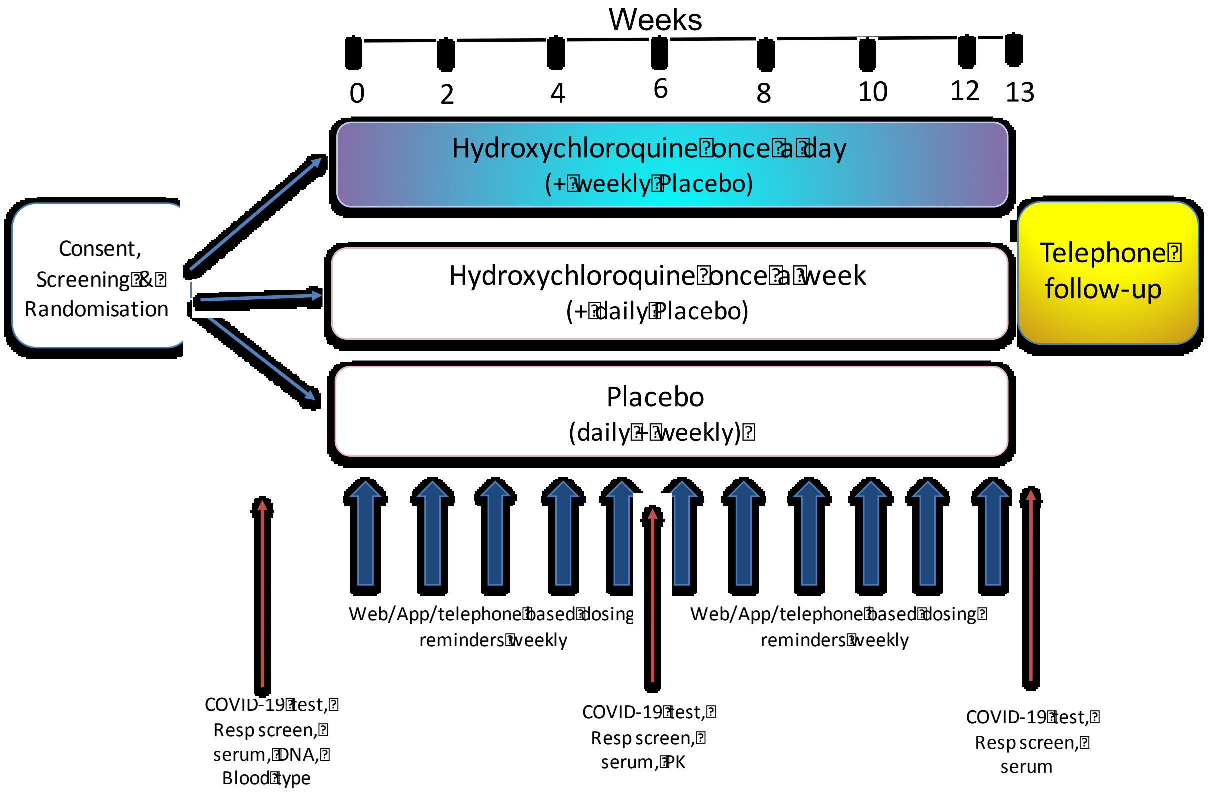

7 Selection and withdrawal of participants

7.1 Inclusion Criteria

To be included in the trial the participant MUST:

1) Have given written informed consent to participate
2) Be aged 18 years to 70 years
3) Not previously have been diagnosed with COVID-19
4) Work in a high-risk secondary or tertiary healthcare setting (hospitals accepting COVID-19 patients) with direct patient-facing care

7.2 Exclusion Criteria

The presence of any of the following will mean participants are ineligible:

1) Known COVID-19 positive test at baseline (if available)
2) Symptomatic for possible COVID-19 at baseline
3) Known hypersensitivity reaction to HCQ, chloroquine or 4-aminoquinolines
4) Known retinal disease
5) Known porphyria
6) Known chronic kidney disease (CKD; eGFR<30ml/min)
7) Known epilepsy
8) Known heart failure or conduction problems
9) Known significant liver disease (Gilbert's syndrome is permitted)
10) Known glucose-6-phosphate dehydrogenase (G6PD) deficiency
11) Currently taking any of the following contraindicated medications:
    a. Digoxin
    b. Chloroquine
    c. Halofantrine
    d. Amiodarone
    e. Moxifloxacin
    f. Cyclosporin
    g. Mefloquine

h. Praziquantel
i. Ciprofloxacin
j. Clarithromycin
k. Prochlorperazine
l. Fluconazole

12) Currently taking hydroxychloroquine or having a clinical indication for taking hydroxychloroquine
13) Currently breastfeeding
14) Unable to be followed-up during the trial
15) Current or future involvement in the active treatment phase of other interventional research studies (excluding observational/non-interventional studies) before study follow-up visit
16) Not able to use or have access to a modern phone device/web-based technology
17) Any other clinical reason which may preclude entry in the opinion of the investigator

Pregnant women are not excluded from participating in the PROLIFIC Trial, on the basis of a moderate amount of data (300-1000 pregnancy outcomes), including from prospective studies, which demonstrate that long-term use of hydroxychloroquine is not associated with increased risk of congenital malformations or poor pregnancy outcomes. In addition, clinically, hydroxychloroquine is used throughout pregnancy at higher doses and for longer periods of time than the proposed trial. Therefore, it is not our intention to exclude pregnancy and pregnancy testing will not be undertaken. In any case, it is unlikely that pregnant healthcare workers will be assigned to areas deemed high-risk for COVID-19 infection.

7.3 Treatment Assignment and Randomisation

Participants who fulfil the eligibility criteria will be randomised to either HCQ dosed every day with weekly placebo, HCQ dosed every week with daily placebo, or placebo dosed daily and weekly. Participants will be randomised by logging into the randomisation website using a secure password. They will enter the following information

- Participant Screening and ID Number
- Participant Initials
- Participant DoB (MM/YYYY)
- Inclusion/Exclusion criteria confirmation (Y/N)

The system will provide a Participant Number and details of the IMP which will be used by the study team to prescribe the treatment allocated in a double-blinded manner. Randomisation will be in a 3:3:2 ratio (HCQ (daily), HCQ (weekly), placebo), using stratified block randomisation. Random block sizes will be used, and stratification will be by study site.

7.4 Withdrawal criteria

7.4.1 Treatment Withdrawal

Participants will remain in the trial but should stop all trial treatments immediately upon the following:

- Diagnosis of COVID-19 via validated NHS testing/other validated test
- Diagnosis of COVID-19 on the basis of clinical symptoms and clinical judgement and/or on advice of treating clinician to stop taking the IMP.

7.4.2 Participant Withdrawal

Primary reasons for withdrawal may include:

- SUSAR or serious adverse event
- Withdrawal of consent - participants may voluntarily withdraw from the study for any reason at any time
- Lost to follow-up
- Participants will be withdrawn at any time if the investigator concludes that it would be in the participant's best interest for any reason.

All of the above are at the discretion of the CI/PI. Reasons for participant withdrawal will be recorded in the Case Report Form (CRF). If any treatment interruption becomes necessary eg due to an SAE, this will be acceptable but participants should be put back on study medication as soon as possible. All study drug interruptions must be recorded in the CRF.

8 Trial Treatments

8.1 Treatment summary

8.1.1 Name and description of IMP

a) Hydroxychloroquine (HCQ)
Hydroxychloroquine sulfate 200 mg tablets (oral administration).
HCQ matching placebo.

8.1.2 Legal Status of IMPs

HCQ is a commercially available prescription-only medicine and is licensed for treatment of malaria as well as lupus and rheumatoid arthritis. Within this trial HCQ is classed as an investigational medicinal product (IMP). A full description is available in the summary of product characteristics (SmPC).
Placebo is unlicensed.

8.1.3 Supply

HCQ and Placebo will be provided free of charge by the sponsors for the duration of the study. IMP will be distributed to all participating sites centrally from Renaclinical Limited. The supply of all IMPs should be ring-fenced at participating sites for this trial in a separate area to non-trial products and records retained in the Pharmacy Site File, noting the location of the storage.
The IMPs will be distributed directly to sites following confirmation that all necessary regulatory, and ethical and site level approvals are in place. Reference should be made to the PROLIFIC pharmacy manual for further details.

8.1.4 Packaging and Labelling

HCQ tablets and matching Placebo will be packed in high density polyethylene bottles with child resistant caps, and will be labelled in accordance with UK regulatory requirements and Good Manufacturing Practice. The sponsor will be responsible for ensuring that the bottles are labelled in line with Directive 2001/20/EC and Medicines for Human Use (Clinical Trials) Regulations 2004 (amended 2006). Please refer to the PROLIFIC Pharmacy Manual for further information.

8.1.5 Storage Conditions

Trial medication will be stored at room temperature (below 25^0C) in its original container in a secure location with access to limited authorised site staff. The storage area will be temperature controlled (i.e. air-conditioning and heating controls available) but temperature readings will not be monitored unless there is cause for concern.

8.1.6 Dispensing

Given the current COVID-19 pandemic, all dispensed stock will be provided to participants by whichever means possible. Where possible, dispensed stock will be collected from the appropriate hospital pharmacy, depending on local trust policy. Stock movement will not be subject to temperature monitoring.

Medication at any dose must be prescribed using a relevant trial prescription.

Documentation of receipt of IMP, prescribing, dispensing and/or destruction of study medication will be maintained for study records.

8.1.7 Treatment duration

The maximum duration of treatment will be approximately 92 days (approximately 13 weeks).

8.1.8 Dose and Administration of IMPs

a) Arm 1: Active Hydroxychloroquine (HCQ) – daily dosing and placebo HCQ - weekly dosing:
Form: Tablets
Route: Oral
Dose and Frequency:
Active HCQ
Days 1-2: Loading phase - 400mg (2 x 200mg tablets) taken TWICE A DAY for 2 days
Days 3 onwards: Maintenance Phase - 200mg (1 x 200mg tablet) taken ONCE A DAY, every day for 90 days (~3 months)

Matched Placebo HCQ
Days 3 onwards: Maintenance Phase - 2 tablets taken ONCE A WEEK on the same day each week (every 7th day) for 90 days (~3 months)

b) Arm 2: Active Hydroxychloroquine (HCQ) - weekly dosing and placebo HCQ – daily dosing:
Form: Tablets
Route: Oral
Dose and Frequency:
Active HCQ
Days 1-2: Loading Phase - 400mg (2 x 200mg tablets) taken TWICE A DAY for 2 days
Days 3 onwards: Maintenance Phase - 400mg (2 x 200mg tablets) taken ONCE A WEEK on the same day each week (every 7th day) for 90 days (~3 months)
Matched Placebo HCQ
Days 3 onwards: Maintenance Phase - 1 tablet taken ONCE A DAY for 90 days (~3 months)

c) Arm 3: Matched placebo Hydroxychloroquine (HCQ) - daily dosing and matched placebo HCQ - weekly dosing
Form: Tablet
Route: Oral
Frequency:
Matched placebo HCQ - daily dosing
Days 1-2: Loading Phase - 2 tablets taken TWICE A DAY for 2 days

Days 3 onwards: Maintenance Phase - 1 tablet taken ONCE A DAY for 90 days (~3 months)

Matched placebo HCQ – weekly dosing

Days 3 onwards: Maintenance Phase - 2 tablets taken ONCE A WEEK on the same day each week (every 7th day) for 90 days (~3 months)

8.1.8.1 Schematic of dosing schedule:

Loading phase (Two days)

Study Arm	Day 1	Day 2
**Daily**	HCQ + HCQ<br>HCQ + HCQ	HCQ + HCQ<br>HCQ + HCQ
**Weekly**	HCQ + HCQ<br>HCQ + HCQ	HCQ + HCQ<br>HCQ + HCQ
**Placebo**	P + P<br>P + P	P + P<br>P + P

Maintenance phase (90 days – 7 day example)

Study Arm	Day 1	Day 2	Day 3	Day 4	Day 5	Day 6	Day 7
**Daily**	HCQ	HCQ	HCQ	HCQ	HCQ	HCQ	HCQ<br>P+P
**Weekly**	P	P	P	P	P	P	P<br>HCQ+HCQ
**Placebo**	P	P	P	P	P	P	P<br>P+P

HCQ = Hydroxychloroquine (200 mg); P = Placebo

8.1.9 Administration

Participants will self-administer the tablets. Each dose should be taken with a meal or glass of milk. Tablets should be swallowed whole.

8.1.10 Missed or replacement doses:

Participants are instructed to not make up vomited doses to maintain the planned dosing schedule. Do not make up for missed doses if more than 12hrs have elapsed after the time it would usually be taken. In the event of missed doses, do not double the dose to make up the missed dose.

Any unused study treatment must be returned to the study site. Unused treatment will then be returned to pharmacy for accountability and will be destroyed. There will be no treatment compliance checks at site level.

8.1.11 Contraindications

As per the latest version of the SmPC for the IMP.

8.1.12 Dosage modifications

Dosage modifications are not permitted in this trial.

8.1.13 Permitted and prohibited medications

Over the counter medications required on an 'as required' basis such as paracetamol (doses <2.0g/day) and other remedies will be permitted. Grapefruit and/or its juice should be avoided. Antacids should be avoided within ~4 hours of dosing.

Contraindicated medications are as follows:

a. Digoxin
b. Chloroquine
c. Halofantrine
d. Amiodarone
e. Moxifloxacin
f. Cyclosporin
g. Mefloquine
h. Praziquantel
i. Ciprofloxacin

j. Clarithromycin
k. Prochlorperazine
l. Fluconazole

8.1.14 Drug interactions

As per the latest version of the SmPC for HCQ.

8.1.15 Method of unblinding

This is a double-blind trial. In order to maintain this blind, study medication will be labelled with a unique Medication Pack Code Number which will be assigned to the subject via the online Sealed Envelope randomisation system. It will not be possible for the study team to determine treatment allocation using the laboratory data during the trial period.

Emergency unblinding: In the event of a valid medical or safety reason, the responsibility to break the treatment code resides solely with the treating clinician (i.e the Investigator or Sub-investigator). Investigators should note that the occurrence of an SAE should not routinely precipitate immediate unblinding.

Sealed Envelope will be used for emergency un-blinding. Appropriately trained and delegated site staff will be given the necessary access rights and permission to access this facility. If un-blinding occurs, the trial medication (HCQ/Placebo) must be discontinued.

9 Schedule of assessments

The following sections describe the schedule of assessments that will be completed by participants. The Schedule of Assessments can be found in Table 1 (section 20.1). Further details are available in the trial procedures manual (TPM).

The first visit will take place at the hospital in which participants are working. Trial specific assessments will only be conducted after the participants have given full written informed consent and have met the inclusion/exclusion criteria.

Eligible participants who provide informed consent will be registered on the study by the trial team. Registered participants will be issued with a unique Screening & Participant ID and this will be used on all trial documentation and samples taken from the participant throughout the trial.

Throughout the trial, the research team may be required to obtain further clinical data from the participant's medical records in order to determine further clinical details for the study.

9.1 Trial assessments (hospital)

9.1.1 Visit 1 - Baseline

- COVID-19 test/s[∞] and respiratory screen*
- Medical and drug history
- Demography & current work setting (i.e. ITU, HDU, ED etc; Area of work, Shift pattern
- Concomitant medications
- Baseline blood sample for blood group, serum store and genetics store[*]
- Demonstration of the app/web/phone-based interface for data entry

∞ = Testing for Covid-19 will be completed whenever possible [currently situation-dependent, however it is anticipated that POC testing will rapidly become widely available and, as such, will be completed when possible]. However, we will not use NHS testing unless current guidance on who should be tested and when, changes, and fits within the protocol;

* = Dependent upon local logistics and NHS resources. Participants may continue with the trial if this measure cannot be taken.

Following completion of baseline assessments, participants will be randomised. Study medication for the first 7 weeks of the trial will be dispensed at this visit (or a prescription issued for the participant to take for dispensing), depending on local (site-specific) arrangements.

9.1.2 Visit 2 – Interim visit

Visit 2 will take place approximately 6 weeks±7 days following the baseline visit. (Ideally, where possible, this visit should be undertaken 6 weeks and 3 days after randomisation, assuming that dosing commences one day following randomisation.
Participants should take their medication after completion of the visit.

- COVID-19 Test/s∞ and respiratory screen
- Review of current work setting (i.e. ITU, HDU, ED etc)
- AE and concomitant medications
- Blood sample for serum store and trough PK*

∞ = Testing for Covid-19 will be completed whenever possible [currently situation-dependent, however it is anticipated that POC testing will rapidly become widely available and, as such, will be completed when possible]. However, we will not use NHS testing unless current guidance on who should be tested and when, changes, and fits within the protocol;

* = Dependent upon local logistics and NHS resources. Participants may continue with the trial if this measure cannot be taken.

Following completion of visit 2, study medication for the remainder of the trial will be dispensed (or a prescription issued for the participant to take for dispensing), depending on local (site-specific) arrangements.

9.1.3 Visit 3

Visit 3 will take place once the participant has finished their medication on the trial.

- COVID-19 Test/s∞ and respiratory screen
- Review of current work setting (i.e. ITU, HDU, ED etc)
- AE and concomitant medications
- Blood sample for serum for serum store*

∞ = Testing for Covid-19 will be completed whenever possible [currently situation-dependent, however it is anticipated that POC testing will rapidly become widely available and, as such, will be completed when possible]. However, we will not use NHS testing unless current guidance on who should be tested and when, changes, and fits within the protocol;

* = Dependent upon local logistics and NHS resources. Participants may continue with the trial if this measure cannot be taken.

9.1.4 Weekly (remote) Assessments

Throughout the course of a subject's participation in the trial the following assessments will be taken weekly using telephone or online web-based / App interface:

- Confirmation of having taken medication (reminder sent daily for first two days then weekly)
- AE check
- Work pattern
- Symptom severity (score)
- Self-reported recovery
- Incidence of health care usage

9.1.5 Ad hoc Assessments (triggered)

At any point, if a trial participant becomes symptomatic, they may be asked to swab themselves (with kit provided) for COVID-19 testing (nasal/throat swab), subject to on-going PHE guidance at the time. If a participant becomes NHS COVID-19 test

positive/COVID-19 positive (on the basis of clinical judgment) during the trial, they will be deemed to have completed the trial in terms of the primary endpoint analysis.

9.2 End of Trial Participation

Participants will have finished their trial participation when they have completed their course of treatment and end of study visit. Participants who become NHS COVID test positive/COVID positive (on the basis of clinical judgment) during the trial will be asked to stop medication at that point.

9.3 Trial restrictions

Participants may not participate if they are taking part in any other interventional clinical trial.

9.4 Procedures and assessments

Below is a brief description of the trial assessments. More detailed information on how to perform the study procedures is listed in the TPM.

a. Test for COVID-19/respiratory screen (nasal/throat swab or blood): Test for SARS-CoV-2 and other common respiratory viruses using validated testing methods available at the time, as per clinical practice guidelines.
b. Baseline genetic blood sample to determine if genetic status predicts susceptibility to infection or response to drug therapy
c. Blood samples (all hospital visits): Blood group$^{\infty}$, trough pk*, serum store (this could include, but is not exclusively limited to tests for, COVID-19 antibodies (IgG, IgM), atypical respiratory serology, CRP, IL-6, D-Dimer, pro-BNP, troponin, ferritin, LDH, Complement C3 and C4)

∞sample for blood group taken at baseline only

*serum for trough pk levels taken on visit 2 only

9.5 Storage of samples

Samples will be stored locally and then eventually transferred to the Cambridge Biomedical campus for analysis.

10 Assessment of Safety

10.1 Definitions

Term	Definition
**Adverse event**	Any untoward medical occurrence in a patient or clinical trial subject administered a medicinal product and which is not necessarily caused by or related to this treatment.
**Adverse Reaction**	All untoward and unintended responses to an investigational medicinal product related to any dose administered. All adverse events judged by either the reporting investigator or the sponsor as having a reasonable causal relationship to a medicinal product qualify as adverse reactions. The expression 'reasonable causal relationship' means to convey, in general, that there is evidence or argument to suggest a causal relationship.
**Unexpected Adverse Reaction**	An adverse reaction, the nature, or severity of which is not consistent with the applicable reference safety information (RSI). When the outcome of the adverse reaction is not consistent with the applicable RSI this adverse reaction should be considered as unexpected.

**Serious Adverse Event (SAE) or Serious Adverse Reaction (SAR)**	Any untoward medical occurrence that at any dose:<br>- results in **death**,<br>- is **life-threatening**<br>- requires **hospitalisation** or **prolongation** of existing inpatient hospitalisation<br>- results in persistent or significant **disability or incapacity**, - is a congenital anomaly or birth defect.<br>- Is another important medical event<br>Life-threatening in the definition of a serious adverse event or serious adverse reaction refers to an event in which the subject was at risk of death at the time of event; it does not refer to an event which hypothetically might have caused death if it were more severe.
**Suspected Unexpected Serious Adverse Reaction (SUSAR)**	A serious adverse reaction, the nature and severity of which is not consistent with the information set out in the RSI.

10.2 Safety monitoring and reporting in the PROLIFIC trial

In the PROLIFIC trial, SAEs will be recorded for all participants as part of the routine trial data collection, however only SARs will be reported to the CI & Sponsor as detailed in section 10.6.

10.3 Reference Safety Information (RSI)

The information used for assessing whether an adverse reaction is expected. This is contained in the summary of product characteristics (SmPC). For this trial, the RSI is section 4.8 for the IMPs listed below.

Name of the Investigational Medicinal Product	SMPC date
Plaquenil-Hydroxychloroquine Suflate 200m Film-coated Tablets, Zentiva Pharma UK Limited	Revision of text: 10th March, 2020

10.4 Expected Adverse Reactions/Serious Adverse Reactions (AR /SARs)

All expected Adverse Reactions are listed in the latest version of the RSI as specified in section 10.2. This must be used when making a determination as to the expectedness of the adverse reaction.

10.5 Expected Adverse Events/Serious Adverse Events (AE/SAE)

The loading dose of Hydroxychloroquine may cause abdominal pain and/or nausea. There are no other expected AEs or SAEs within this trial.

10.6 Recording and Evaluating SAEs, Reporting SARs

The Principal Investigators are responsible for recording all SAEs in the trial data collection tools, ensuring that the assessment of all SAEs for relatedness and expectedness is completed and the onward notification of all SARs to the Chief Investigator and Sponsor immediately but not more than 24 hours of first notification. A further review of expectedness will be undertaken by the Chief Investigator. The sponsor has to keep detailed records of all SARs reported to them by the trial team.

The Chief Investigator is also responsible for prompt reporting of all Serious Adverse Reactions to the competent authority (e.g. MHRA) of each concerned Member State if they could:

- adversely affect the health of participants
- impact on the conduct of the trial
- alter the competent authority's authorization to continue the trial in accordance with Directive 2001/20/EC

The completed SAR form can be faxed or emailed. Details of where to report the SARs can be found on the PROLIFIC SAR form and the front cover of the protocol.

10.6.1 Assessment of seriousness

Seriousness is assessed against the criteria in section 10.1. This defines whether the event is an adverse event, serious adverse event or a serious adverse reaction

10.6.2 Assessment of causality

Definitely: A causal relationship is clinically/biologically certain. **This is therefore an Adverse Reaction**

Probably: A causal relationship is clinically / biologically highly plausible and there is a plausible time sequence between onset of the AE and administration of the investigational medicinal product and there is a reasonable response on withdrawal. **This is therefore an Adverse Reaction.**

Possibly: A causal relationship is clinically / biologically plausible and there is a plausible time sequence between onset of the AE and administration of the investigational medicinal product. **This is therefore an Adverse Reaction.**

Unlikely: A causal relation is improbable and another documented cause of the AE is most plausible. **This is therefore an Adverse Event.**

Unrelated: A causal relationship can be definitely excluded and another documented cause of the AE is most plausible. **This is therefore an Adverse Event.**

Unlikely and unrelated causalities are considered NOT to be trial drug related. Definitely, probably and possibly causalities are considered to be trial drug related.

A pre-existing condition must not be recorded as an SAE unless the condition worsens during the trial and meets the criteria for reporting or recording in the appropriate section of the CRF.

10.6.3 Clinical assessment of severity

Mild: The participant is aware of the event or symptom, but the event or symptom is easily tolerated

Moderate: The participant experiences sufficient discomfort to interfere with or reduce his or her usual level of activity

Severe: Significant impairment of functioning; the participant is unable to carry out usual activities and / or the participant's life is at risk from the event.

10.6.4 Recording of adverse events

Adverse events and adverse reactions and Serious Adverse Events should be recorded in the medical notes and the appropriate section of the CRF and/or AE/AR log.

Serious Adverse Reactions must be reported to the sponsor as detailed in section 10.6.

10.7 Reporting of Suspected Unexpected Serious Adverse Reactions (SUSARs)

All suspected adverse reactions which occur in the PROLIFIC trial, and that are both unexpected and serious (SUSARs) are subject to expedited reporting. Please see section 10.3 for the Reference Safety Information to be used in this trial.

10.7.1 Who should report and whom to report to?

The Sponsor delegates the responsibility of notification of SUSARs to the Chief Investigator. The Chief Investigator must report all the relevant safety information previously described, to the:

- Sponsor
- competent authorities in the concerned member states (e.g. MHRA)
- Ethics Committee in the concerned member states

The Chief Investigator shall inform all investigators concerned of relevant information about SUSARs that could adversely affect the safety of participants.

10.7.2 When to report SUSARs?

Fatal or life-threatening SUSARs

All parties listed in section 10.7.1 must be notified as soon as possible but no later than **7 calendar days** after the trial team and Sponsor has first knowledge of the minimum criteria for expedited reporting.

In each case relevant follow-up information should be sought and a report completed as soon as possible. It should be communicated to all parties within an additional **8 calendar days**.

Non-fatal and non-life-threatening SUSARs

All other SUSARs and safety issues must be reported to all parties listed in section 10.7.1 as soon as possible but no later than **15 calendar days** after first knowledge of the minimum criteria for expedited reporting. Further relevant follow-up information should be given as soon as possible.

10.7.3 How to report SUSARs?

Minimum criteria for initial expedited reporting of SUSARs

Information on the final description and evaluation of an adverse reaction report may not be available within the required time frames for reporting. For regulatory purposes, initial expedited reports should be submitted within the time limits as soon as the minimum following criteria are met:

a) a suspected investigational medicinal product

b) an identifiable participant (e.g. trial participant code number)

c) an adverse event assessed as serious and unexpected, and for which there is a reasonable suspected causal relationship

d) an identifiable reporting source

and, when available and applicable:

- a unique clinical trial identification (EudraCT number or in case of non-European Community trials the sponsor's trial protocol code number)
- a unique case identification (i.e. sponsor's case identification number)

Follow-up reports of SUSARs

In case of incomplete information at the time of initial reporting, all the appropriate information for an adequate analysis of causality should be actively sought from the reporter or other available sources. Further available relevant information should be reported as follow-up reports.

In certain cases, it may be appropriate to conduct follow-up of the long-term outcome of a particular reaction.

Format of the SUSAR reports

Electronic reporting is the expected method for expedited reporting of SUSARs to the competent authority. The format and content as defined by the competent authority should be adhered to.

10.8 Pregnancy Reporting

All pregnancies within the trial (either the trial participant or the participant's partner) should be reported to the Chief Investigator and the Sponsor using the relevant Pregnancy Reporting Form within 24 hours of notification.

Pregnancy is not considered an AE unless a negative or consequential outcome is recorded for the mother or child/fetus. If the outcome meets the serious criteria, this would be considered an SAE.

11 Statistics

11.1 Statistical methods

The primary endpoint, time to disease onset, will be compared using a Cox proportional hazards model, adjusting with fixed effects for

- Age group (18-49, 50-59, 60+)
- Sex
- Clinical area (high, medium and low COVID-19 exposure risk)
- Site
- Known high-risk pre-existing conditions (e.g. CVD, HT, DM, cancer, lung disease, immunosuppressed individuals), vs not.

Estimates, 95% confidence intervals and p-values for the hazard ratios will be provided.

The primary comparison will be the placebo arm versus the pooled arms of HCQ dosed daily and HCQ dosed weekly. If, and only if, this primary comparison is statistically significant at a 5% significance level, will a further comparison will be made between the two active arms (HCQ dosed daily versus HCQ dosed weekly). Pooling the two HCQ arms in a comparison with placebo will be sufficiently powered and is interpretable.

11.2 Number of Participants to be enrolled

Assuming a hazard ratio of 0.7 and an infection rate of 0.3 at 3 months in the placebo arm, 261 events will provide 80% power under a 2-sided 5% significance level for the primary analysis. Assuming the recruitment period is very rapid and a conservative follow-up of 3 months, recruiting 250 placebo participants and 750 HCQ participants (1000 in total), will provide the required number of events. The aim is to recruit 1200 participants to account for dropouts.

11.3 Interim analysis

Interim analyses will be scheduled based on regular calendar time intervals, to give flexibility to adapt to external information. DMC meetings will occur regularly, with the ability to amend the frequency of meetings. The first interim analysis will be conducted at approximately 6 weeks after the first participant has been recruited, to assess recruitment and event rates. Interim analysis will also be recommended at a time shortly before recruitment is scheduled to be halted, to assess if the sample size assumptions are realistic.

The primary endpoint and primary comparison will be sequentially analysed at each interim analysis using a Lan-DeMets error-spending approach corresponding to symmetric 2-sided O'Brien-Fleming boundaries (**https://doi.org/10.1002/sim.4780131308**). An overall 5% significance level will be preserved. The unit of information is defined as the proportion of events observed relative to the total target number of events (261). The assumptions regarding the sample size (including the event rate and recruitment rate) may be reviewed and the total sample size and target number of events may be adapted. Conditional and Predictive power calculations may be used to inform any such sample size adaptations. The overall significance level will be preserved by spending the remaining error, following any preceding interim analyses, using a revised definition of information based on a revised target number of events.

11.4 Criteria for the premature termination of the trial

If the sequential interim analyses suggest a large positive, or negative, effect then the DMC may consider recommending early termination.

11.5 Definition of the end of the trial

The end of trial will be the date of the last participant's last visit.

12 Data handling and record keeping

All data will be transferred into a Case Report Form (CRF) which will be anonymised. All trial data in the CRF must be extracted from and be consistent with the relevant source documents. The CRFs must be completed, dated and signed by the investigator or designee in a timely manner. It remains the responsibility of the investigator for the timing, completeness, legibility and accuracy of the CRF pages. The CRF will be accessible to trial coordinators, data managers, the Investigators, Clinical Trial Monitors, Auditors and Inspectors as required.

Completed CRFs should be emailed to the trial coordination centre (using nhs.net email; cambs.cardiovascular@nhs.net. The investigators must ensure that trial related documentation sent to the trial coordination centre contains no patient identifiable data. A trial specific data management plan will describe in detail the data management processes using the CRF and the trial database.

All CRF pages must be clear, legible and completed in black ink. Any errors should be crossed with a single stroke so that the original entry can still be seen. Corrections should be inserted and the change dated and initialled by the investigator or designee. If it is not clear why the change has been made, an explanation should be written next to the change. Typing correction fluid must not be used.

12.1 Source Data

To enable peer review, monitoring, audit and/or inspection the investigator must agree to keep records of all participants (sufficient information to link records e.g., CRFs, hospital records and samples), all original signed informed consent forms and copies of the CRF pages.

Source data may include but is not limited to:

- Informed Consent Form
- Relevant sections of the Case Report Form (written or electronic), as defined by the TPM
- On-line or telephone-based weekly questionnaires
- Medical Records (written or electronic)

• On-line laboratory test results systems

12.2 Data Protection & Participant Confidentiality

All investigators and trial site staff involved in this trial must comply with the requirements of the Data Protection Act 2018 and Trust Policy with regards to the collection, storage, processing and disclosure of personal information and will uphold the Act's core principles.

13 Data Monitoring Committee

The DMC will be comprised of an unblinded independent group, as defined in a separate charter document, which will define the role of the DMC. The DMC will be responsible for the review of all safety (but not exploratory) data and will meet regularly whilst the trial is ongoing, from opening to recruitment until the final visit of the last participant. Additionally, ad hoc DMC meetings may be triggered if conditions are met as specified in section 11.3.

14 Ethical & Regulatory considerations

14.1 Consent

The informed consent form must be approved by the REC and must be in compliance with GCP, local regulatory requirements and legal requirements. The investigator must ensure that each trial participant is fully informed about the nature and objectives of the trial and possible risks associated with their participation.

The investigator will obtain written informed consent from each participant before any trial-specific activity is performed. The informed consent form used for this trial and any change made during the course of this trial, must be prospectively approved by the REC. The investigator will retain the original of each participant's signed informed consent form.

Any new information which becomes available, which might affect the participant's willingness to continue participating in the trial will be communicated to the participant as soon as possible. We will provide this information verbally over the telephone or at their next visit.

14.2 Ethical committee review

Before the start of the trial or implementation of any amendment we will obtain approval of the trial protocol, protocol amendments, informed consent forms and other relevant documents e.g., advertisements and GP information letters if applicable from the REC. All correspondence with the REC will be retained in the Trial Master File/Investigator Site File. Annual reports will be submitted to the REC in accordance with national requirements. It is the Chief Investigator's responsibility to produce the annual reports as required.

14.3 Regulatory Compliance

The trial will not commence until a Clinical Trial Authorisation (CTA) is obtained from the MHRA. The protocol and trial conduct will comply with the Medicines for Human Use (Clinical Trials) Regulations 2004 and any relevant amendments. Development Safety Update Reports (DSURs) will be submitted to the MHRA in accordance with national requirements. It is the Chief Investigator's responsibility to produce these reports as required.

14.4 Protocol Amendments

Protocol amendments must be reviewed and agreement received from the Sponsor for all proposed amendments prior to submission to the REC and/or MHRA. The only circumstance in which an amendment may be initiated prior to REC and/or MHRA approval is where the change is necessary to eliminate apparent, immediate risks to the participants (Urgent Safety Measures). In this case, accrual of new participants will be halted until the REC and/or MHRA approval has been obtained.

14.5 Peer Review

The PROLIFIC trial protocol has been peer-reviewed by the Evolution Educational Trust, who is separate to the sponsor and the CUH COVID-19 Trials Prioritisation Group.

14.6 Declaration of Helsinki and Good Clinical Practice

The trial will be performed in accordance with the spirit and the letter of the declaration of Helsinki, the conditions and principles of good clinical practice, the protocol and applicable local regulatory requirements and laws.

14.7 GCP Training

All trial staff must hold evidence of appropriate GCP training or undergo GCP training prior to undertaking any responsibilities on this trial. This training should be updated every 2 years or in accordance with local Trust policies.

15 Sponsorship, Financial and Insurance

The trial is sponsored by Cambridge University Hospitals NHS Foundation Trust. The study will be funded by the Evolution Education Trust.

Cambridge University Hospitals NHS Foundation Trust, as a member of the NHS Clinical Negligence Scheme for Trusts, will accept full financial liability for harm caused to participants in the clinical trial caused through the negligence of its employees and honorary contract holders. There are no specific arrangements for compensation should a participant be harmed through participation in the trial, but no-one has acted negligently.

16 Monitoring, Audit & Inspection

The investigator must make all trial documentation and related records available should an MHRA Inspection occur. Should a monitoring visit or audit be requested, the investigator must make the trial documentation and source data available to the Sponsor’s representative. All patient data must be handled and treated confidentially.

The Sponsor’s monitoring frequency will be determined by an initial risk assessment performed prior to the start of the trial. A detailed monitoring plan will be generated detailing the frequency and scope of the monitoring for the trial. Throughout the course of the trial, the risk assessment will be reviewed and the monitoring frequency adjusted as necessary.

On-site or remote monitoring will be conducted for all participating sites. The scope and frequency of the monitoring will be determined by the risk assessment and detailed in the Monitoring Plan for the trial.

17 Protocol Compliance and Breaches of GCP

Prospective, planned deviations or waivers to the protocol are not allowed under the UK regulations on Clinical Trials and must not be used. Protocol deviations, non-compliances, or breaches are departures from the approved protocol. They can happen

at any time, but are not planned. They must be adequately documented on the relevant forms and reported to the Chief Investigator and Sponsor immediately. Deviations from the protocol which are found to occur constantly again and again will not be accepted and will require immediate action and could potentially be classified as a serious breach. Any potential/suspected serious breaches of GCP must be reported immediately to the Sponsor without any delay.

18 Publications policy

Ownership of the data arising from this trial resides with the Sponsor and their delegates. On completion of the trial the data will be tabulated and analysed and a final trial report prepared.

19 References

1. Remuzzi A, Remuzzi G. COVID-19 and Italy: what next? *Lancet*. 2020.
2. Ran L, Chen X, Wang Y, Wu W, Zhang L, Tan X. Risk Factors of Healthcare Workers with Corona Virus Disease 2019: A Retrospective Cohort Study in a Designated Hospital of Wuhan in China. *Clin Infect Dis*. 2020.
3. Zhu N, Zhang D, Wang W, Li X, Yang B, Song J, Zhao X, Huang B, Shi W, Lu R, Niu P, Zhan F, Ma X, Wang D, Xu W, Wu G, Gao GF, Tan W, China Novel Coronavirus I, Research T. A Novel Coronavirus from Patients with Pneumonia in China, 2019. *N Engl J Med*. 2020;382:727-733.
4. Gautret P, Lagier J, Parola P, Hoang V. Hydroxychloroquine and azithromycin as a treatment of COVID-19: results of an open label non-randomized clinical trial. *International Journal of Antimicrobial Agents*. 2020.
5. Wang M, Cao R, Zhang L, Yang X, Liu J, Xu M, Shi Z, Hu Z, Zhong W, Xiao G. Remdesivir and chloroquine effectively inhibit the recently emerged novel coronavirus (2019-nCoV) in vitro. *Cell Res*. 2020;30:269-271.
6. Biot C, Daher W, Chavain N, Fandeur T, Khalife J, Dive D, De Clercq E. Design and synthesis of hydroxyferroquine derivatives with antimalarial and antiviral activities. *J Med Chem*. 2006;49:2845-9.
7. Yao X, Ye F, Zhang M, Cui C, Huang B, Niu P, Liu X, Zhao L, Dong E, Song C, Zhan S, Lu R, Li H, Tan W, Liu D. In Vitro Antiviral Activity and Projection of Optimized Dosing Design of Hydroxychloroquine for the Treatment of Severe Acute Respiratory Syndrome Coronavirus 2 (SARS-CoV-2). *Clin Infect Dis*. 2020.
8. Fox RI. Mechanism of action of hydroxychloroquine as an antirheumatic drug. *Semin Arthritis Rheum*. 1993;23:82-91.
9. Lim HS, Im JS, Cho JY, Bae KS, Klein TA, Yeom JS, Kim TS, Choi JS, Jang IJ, Park JW. Pharmacokinetics of hydroxychloroquine and its clinical implications in chemoprophylaxis against malaria caused by Plasmodium vivax. *Antimicrob Agents Chemother*. 2009;53:1468-75.

20 Appendices

20.1 Table 1 Schedule of assessments and procedures

Assessments	Trial Assessments (Hospital)			Remote
	Visit 1 Baseline	Visit 2 Interim	Visit 3 End of study	Online Assessments weekly
**Attend Hospital clinic**				
Informed consent and Inclusion/Exclusion criteria	X			
COVID-19 Test/s[a]	X	X	X	
Respiratory screen[a,b]	X	X	X	
Medical/ Drug History	X			
Demography	X			
Current work setting	X	X	X	X
Adverse event check	X	X	X	X
Concomitant medication check	X	X	X	
Blood group check[b]	X			
Trough pk[b]		X		
Serum store[b]	X	X	X	
Genetics store[b]	X			
Randomisation	X			
Study medication dispensed	X	X		
**Online App/Web-based interface**				
Symptom severity	X	X	X	X
self-reported recovery	X	X	X	X
Incidence of healthcare usage self-reported recovery	X	X	X	X
IMP dosing compliance check	X	X	X	X

a. Test as used clinically in local site. However, we will not use NHS testing unless current guidance on who should be tested and when, changes, and fits within the protocol

b. Dependent on local logistics and NHS resources. Participants may continue with the trial if this measure cannot be taken.

20.2 Pharmacokinetic simulations to support Hydroxychloroquine dosing

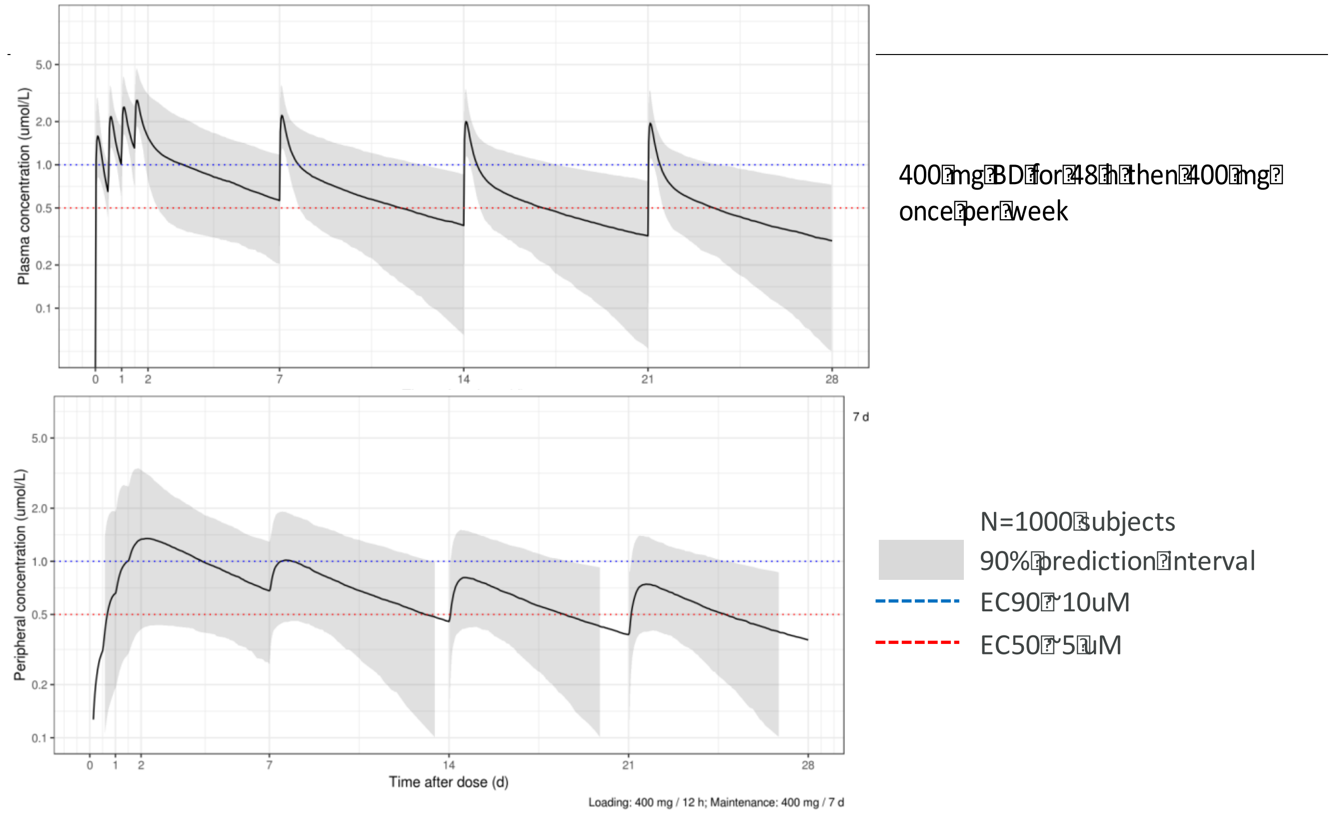

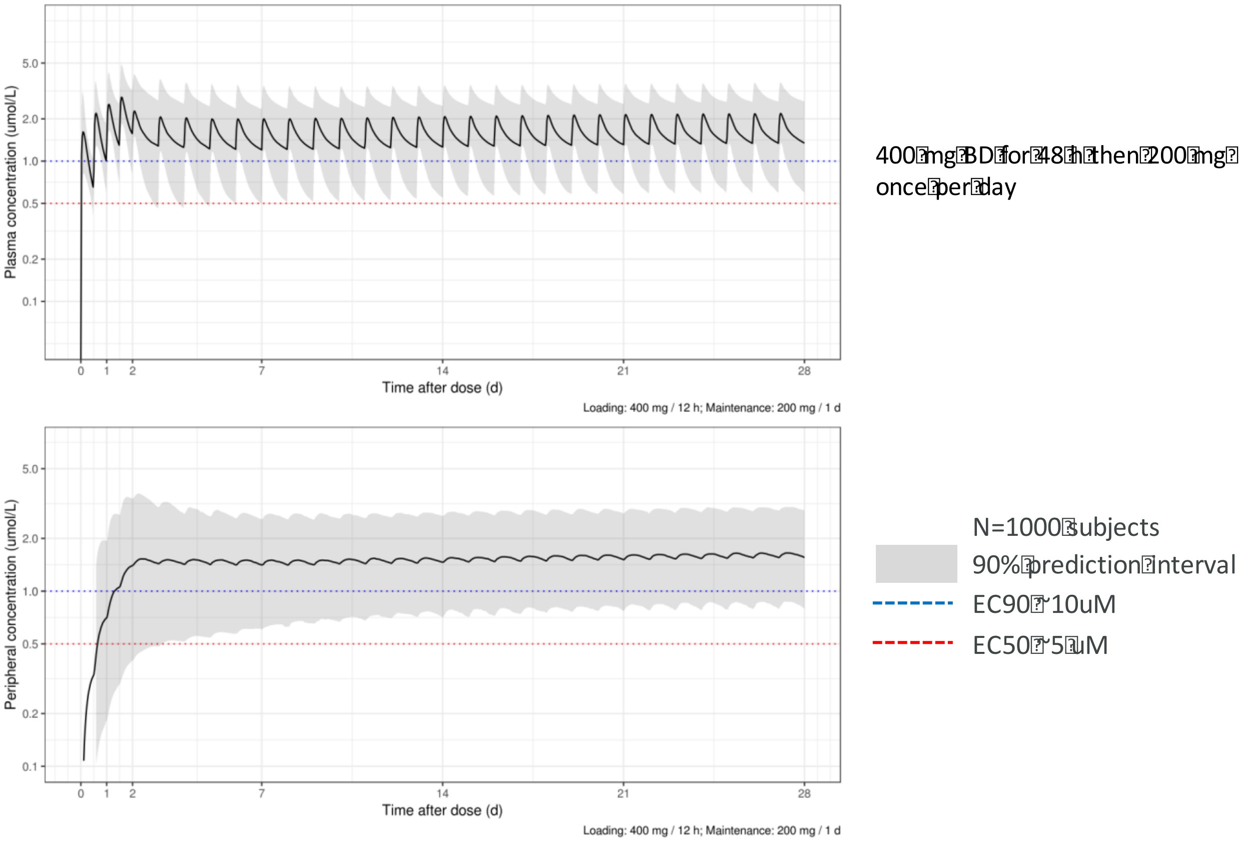

PK simulation data based on[7, 9] and provided by Clinical Pharmacology and Quantitative Pharmacology, Astra Zeneca.
