# Supplementary material for: ChemoPROphyLaxIs with hydroxychloroquine For covId-19 infeCtious disease (PROLIFIC) to prevent covid-19 infection in frontline healthcare workers: A structured summary of a study protocol for a randomised controlled trial
Source: Trials. 2020 Jul 2;21:604. doi: 10.1186/s13063-020-04543-4 (PMC7330261; doi:10.1186/s13063-020-04543-4)
Supplement: Supplementary file 2 — Additional file 2. [file 13063_2020_4543_MOESM2_ESM.docx]

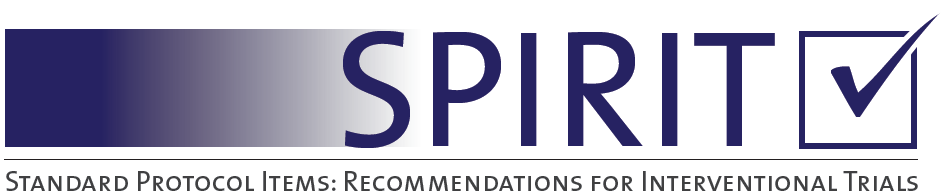


SPIRIT 2013 Checklist: Recommended items to address in a clinical trial protocol and related documents*

| Section/item | ItemNo | Description |
| --- | --- | --- |
| **Administrative information** | | |
| Title | 1 | Descriptive title identifying the study design, population, interventions, and, if applicable, trial acronym **YES** |
| Trial registration | 2a | Trial identifier and registry name. If not yet registered, name of intended registry **YES** |
|  | 2b | All items from the World Health Organization Trial Registration Data Set **(Clinical Trials.gov ID NCT04352933)** |
| Protocol version | 3 | Date and version identifier **YES** |
| Funding | 4 | Sources and types of financial, material, and other support  The trial is sponsored by Cambridge University Hospitals NHS Foundation Trust. This study is supported by the Cambridge NIHR Comprehensive BRC. |
| Roles and responsibilities | 5a | Names, affiliations, and roles of protocol contributors **-see protocol** |
|  | 5b | Name and contact information for the trial sponsor  **Cambridge University Hospitals NHS Foundation Trust**  **Hills Road, Cambridge**  **CB2 0QQ** |
|  | 5c | Role of study sponsor and funders, if any, in study design; collection, management, analysis, and interpretation of data; writing of the report; and the decision to submit the report for publication, including whether they will have ultimate authority over any of these activities  **The Sponsor (Cambridge University Hospitals NHS Foundation Trust) will undertake a risk assessment of the trial and scope and frequency of the monitoring will be determined by the risk assessment and detailed in the Monitoring Plan for the trial.** |
|  | 5d | Composition, roles, and responsibilities of the coordinating centre, steering committee, endpoint adjudication committee, data management team, and other individuals or groups overseeing the trial, if applicable (see Item 21a for data monitoring committee)  **This trial will be managed by the Cambridge Clinical Trials Unit (CCTU). The trial steering committee includes protocol contributors as listed in the protocol. CCTU teams will provide the infrastructure for the trial including data management team, statistical analysis and IT infrastructure.** |
| Introduction |  |  |
| Background and rationale | 6a | Description of research question and justification for undertaking the trial, including summary of relevant studies (published and unpublished) examining benefits and harms for each intervention  The rationale for the PROLIFIC trial is to determine if chemoprophylaxis with hydroxychloroquine can reduce the risk of infection by SARS-CoV-2 (severe acute respiratory distress syndrome coronavirus-2) and associated COVID-19 disease, in frontline healthcare workers.  The justification for undertaking this trial is the current global pandemic of COVID-19, potential for subsequent pandemic waves of this infection, the excess healthcare burden and mortality associated with COVID-19, and importantly, the fact healthcare workers are at higher risk of acquiring this life-threatening infection, given the nature of their interactions and close proximity to patients with COVID-19 (1-5).  Currently there is no vaccine against SARS-CoV-2 infection and there are no specific drugs which are proven to prevent, treat or delay the progression of COVID-19 in at-risk groups. In this context, repurposing of licensed drugs for novel use in COVID-19, maybe a useful strategy for prevention and treatment of the disease, because knowledge of safety profiles, side effects and drug interactions are already well recognised.  Hydroxychloroquine is a widely used anti-malarial drug with immunomodulatory effects that is also used to treat a number of rheumatological conditions. *In vitro* data in a previous SARS outbreak, showed that hydroxychloroquine has anti-viral activity against SARS-CoV-1 (6). In addition, preliminary observational data describes a possible benefit of hydroxychloroquine in the treatment of patients with COVID-19 (7). However, a more recent study which is *In Press*, showed no benefit of hydroxychloroquine in the treatment of established COVID-19 disease (8), although treatment was initiated late on in the disease course.  Interestingly, an *in vitro* study of SARS-CoV-2 infected Vero cells, showed that hydroxychloroquine exhibited an anti-viral effect that was superior to chloroquine (also being considered for therapeutic use in COVID-19) when administered *prior* to viral challenge (9), suggesting a possible role as an effective chemoprophylactic agent against COVID-19. Although the molecular mechanisms for hydroxychloroquine’s potential therapeutic effects have not been fully elucidated, available data suggest that inhibition of SARS-CoV-2 infection may occur by a number of plausible mechanisms. These include prevention of acidification of the endosomal compartment, which reduces or prevents fusion of the SARS-CoV-2 envelope with the endosomal membrane; and inhibition of glycosylation of viral proteins and other processes, resulting in an anti-viral effect of the drug (9). As such, treatment with hydroxychloroquine may create a milieu in which the virus is unable to replicate effectively and cause progression of COVID-19 disease.  As yet, there are no *in vivo* data in humans evaluating the potential chemoprophylactic effects of hydroxychloroquine against SARS-CoV-2 and development of COVID-19. Such data are now urgently required from high quality, placebo-controlled randomised trials. |
|  |  | Hydroxychloroquine is a well-tolerated, commonly used drug, and its pharmacokinetic profile is well understood (10, 11). Based on its promising *in vitro* anti-viral data against SARS-Cov-2, hydroxychloroquine is an ideal drug to re-purpose and trial as a chemoprophylaxis treatment for COVID-19. The rationale for doses of hydroxychloroquine used in this particular study are based on our simulated pharmacokinetic modelling (see protocol appendix), and are lower than hydroxychloroquine doses used in licensed treatments. Therefore risk of dose related side effects are also likely reduced.  This double-blind, randomised placebo-controlled trial is crucially important to enable rigorous evaluation of the potential role of hydroxychloroquine as a chemoprophylaxis agent for COVID-19 in healthcare workers who are likely to be exposed to SARs-CoV-2 in healthcare settings.  1.Coronavirus disease 2019 (COVID-19) in the EU/EEA and the UK – eighth update. Accessed on 22^nd^ April at: https://www.ecdc.europa.eu/sites/default/files/documents/covid-19-rapid-risk-assessment-coronavirus-disease-2019-eighth-update-8-april-2020.pdf  2. Remuzzi A, Remuzzi G. COVID-19 and Italy: what next? Lancet. 2020  3. Xu S, Li Y. Beware second wave of COVID-19. Lancet 2020. Accessed on 22^nd^ April at: https://doi.org/10.1016/ S0140-6736(20)30845-X  4. Ran L, Chen X, Wang Y, Wu W, Zhang L, Tan X. Risk Factors of Healthcare Workers with Corona Virus Disease 2019: A Retrospective Cohort Study in a Designated Hospital of Wuhan in China. Clin Infect Dis. 2020.  5. Zhu N, Zhang D, Wang W, Li X, Yang B, Song J, Zhao X, Huang B, Shi W, Lu R, Niu P, Zhan F, Ma X, Wang D, Xu W, Wu G, Gao GF, Tan W, China Novel Coronavirus I, Research T. A Novel Coronavirus from Patients with Pneumonia in China, 2019. N Engl J Med. 2020;382:727-733.  6. Biot C, Daher W, Chavain N, Fandeur T, Khalife J, Dive D, De Clercq E. Design and synthesis of hydroxyferroquine derivatives with antimalarial and antiviral activities. J Med Chem. 2006;49:2845-9.  7. Gautret P, Lagier J, Parola P, Hoang V. Hydroxychloroquine and azithromycin as a treatment of COVID-19: results of an open label non-randomized clinical trial. International Journal of Antimicrobial Agents. 2020.  8. Tang W, Cao Z, Han M, Wang Z, Chen J, Sun W et al. Hydroxychloroquine in patients with COVID-19: an open-label, randomized controlled trial. Accessed on 22^nd^ April at: https://www.medrxiv.org/content/10.1101/2020.04.10.20060558v1  9. Yao X, Ye F, Zhang M, Cui C, Huang B, Niu P, Liu X, Zhao L, Dong E, Song C, Zhan S, Lu R, Li H, Tan W, Liu D. In Vitro Antiviral Activity and Projection of Optimized Dosing Design of Hydroxychloroquine for the Treatment of Severe Acute Respiratory Syndrome Coronavirus 2 (SARS-CoV-2). Clin Infect Dis. 2020.  10. Fox RI. Mechanism of action of hydroxychloroquine as an antirheumatic drug. Semin Arthritis Rheum. 1993;23:82-91.  11. Lim HS, Im JS, Cho JY, Bae KS, Klein TA, Yeom JS, Kim TS, Choi JS, Jang IJ, Park JW. Pharmacokinetics of hydroxychloroquine and its clinical implications in chemoprophylaxis against malaria caused by Plasmodium vivax. Antimicrob Agents Chemother. 2009;53:1468-75. |
|  | 6b | Explanation for choice of comparators  This double-blind (participant and investigator) placebo-controlled randomised trial will have three comparator arms. Treatment period from randomisation is approximately 13 weeks.  A) Hydroxychloroquine daily dosing (+ weekly placebo)  B) Hydroxychloroquine weekly dosing (+ daily placebo)  C) Hydroxychloroquine-identical appearance matched placebo dosing (daily and weekly)  All trial arms will have the same dosing schedule and number of tablets to take over the course of the study, although the overall dose of hydroxychloroquine between the two active arms will be different. This is explained in more detail in section 11a.  The rationale for this approach is the need for a placebo arm to evaluate for efficacy (or harm) of hydroxychloroquine. The different dosing regimens of hydroxychloroquine are based on simulated pharmacokinetic modelling of both daily vs weekly hydroxychloroquine to estimate peripheral blood concentration of hydroxychloroquine required for anti-viral activity based on published *in vitro* data. |
| Objectives | 7 | Specific objectives or hypotheses  **Primary objective:** To determine whether chemoprophylaxis with hydroxychloroquine versus placebo increases time to COVID-19 disease in frontline healthcare workers.  **Secondary objectives**   1. To determine whether chemoprophylaxis with daily versus weekly dosing of increases time to COVID-19 disease compared with placebo, in frontline healthcare workers. 2. To compare the number of COVID-19 cases between each trial arm 3. To compare the percentage of COVID-19 positive individuals with current testing methods versus serologically-proven COVID-19 in each trial arm 4. To compare COVID-19 disease severity in each trial arm 5. To compare recovery time from COVID-19 infection in each trial arm |
| Trial design | 8 | Description of trial design including type of trial (eg, parallel group, crossover, factorial, single group), allocation ratio, and framework (eg, superiority, equivalence, noninferiority, exploratory)  **Trial design:** double blind (participant and investigator) placebo controlled trial.  Allocation ratio to treatment is 3:3:2 for hydroxychloroquine daily dosing vs hydroxychloroquine weekly dosing vs matched placebo tablets.  Framework is superiority of active treatment hydroxychloroquine compared with placebo. |
| Methods: Participants, interventions, and outcomes | | |
| Study setting | 9 | Description of study settings (eg, community clinic, academic hospital) and list of countries where data will be collected. Reference to where list of study sites can be obtained  National Health Service hospitals managing patients with COVID-19  Country: England/UK  Sites- Cambridge University hospital s NHS Foundation Trust, further sites awaiting confirmation |
| Eligibility criteria | 10 | Inclusion and exclusion criteria for participants. If applicable, eligibility criteria for study centres and individuals who will perform the interventions (eg, surgeons, psychotherapists)  **Inclusion criteria:**  To be included in the trial the participant MUST:   1. Have given written informed consent to participate 2. Be aged 18 years to 70 years 3. Not previously have been diagnosed with COVID-19 4. Work in a high-risk secondary or tertiary healthcare setting (hospitals accepting COVID-19 patients) with direct patient-facing care   **Exclusion criteria:**  The presence of any of the following will mean participants are ineligible:   1. Known COVID-19 positive test at baseline (if available) 2. Symptomatic for possible COVID-19 at baseline 3. Known hypersensitivity reaction to hydroxychloroquine, chloroquine or 4-aminoquinolines 4. Known retinal disease 5. Known porphyria 6. Known chronic kidney disease (CKD; eGFR<30ml/min) 7. Known epilepsy 8. Known heart failure or conduction problems 9. Known significant liver disease (Gilbert’s syndrome is permitted) 10. Known glucose-6-phosphate dehydrogenase (G6PD) deficiency 11. Currently taking any of the following contraindicated medications: Digoxin, Chloroquine, Halofantrine, Amiodarone, Moxifloxacin, Cyclosporin, Mefloquine, Praziquantel, Ciprofloxacin, Clarithromycin, Prochlorperazine, Fluconazole 12. Currently taking hydroxychloroquine or having a clinical indication for taking hydroxychloroquine 13. Currently breastfeeding 14. Unable to be followed-up during the trial 15. Current or future involvement in the active treatment phase of other interventional research studies (excluding observational/non-interventional studies) before study follow-up visit 16. Not able to use or have access to a modern phone device/web-based technology 17. Any other clinical reason which may preclude entry in the opinion of the investigator |
| Interventions | 11a | Interventions for each group with sufficient detail to allow replication, including how and when they will be administered  **Arm A:** Active Hydroxychloroquine (– daily dosing and placebo-matched hydroxychloroquine) - weekly dosing. Form: Tablets  Route: Oral. Dose and Frequency:  Active hydroxychloroquine:  Days 1-2: Loading phase - 400mg (2 x 200mg tablets) taken twice a day for 2 days  Days 3 onwards: Maintenance Phase - 200mg (1 x 200mg tablet) taken once daily, every day for 90 days (~3 months)  Matched Placebo hydroxychloroquine:  Days 3 onwards: Maintenance Phase - 2 tablets taken once a week on the same day each week (every 7^th^ day) for 90 days (~3 months)  **Arm B:** Active Hydroxychloroquine - weekly dosing and placebo matched hydroxychloroquine – daily dosing. Form: Tablets  Route: Oral. Dose and Frequency:  Active hydroxychloroquine:  Days 1-2: Loading Phase - 400mg (2 x 200mg tablets) taken twice daily for 2 days  Days 3 onwards: Maintenance Phase - 400mg (2 x 200mg tablets) taken once a week on the same day each week (every 7^th^ day) for 90 days (~3 months)  Matched Placebo hydroxychloroquine:  Days 3 onwards: Maintenance Phase - 1 tablet taken once daily for 90 days (~3 months)  **Arm C:** Matched placebo Hydroxychloroquine - daily dosing and matched placebo hydroxychloroquine - weekly dosing. Form: Table. Route: Oral. Frequency:  Matched placebo hydroxychloroquine - daily dosing:  Days 1-2: Loading Phase - 2 tablets taken twice daily for 2 days  Days 3 onwards: Maintenance Phase - 1 tablet taken once daily for 90 days (~3 months)  Matched placebo hydroxychloroquine – weekly dosing:  Days 3 onwards: Maintenance Phase - 2 tablets taken once a week on the same day each week (every 7th day) for 90 days (~3 months) Administration: Participants will self-administer the tablets. Each dose should be taken with a meal or glass of milk. Tablets should be swallowed whole. **Schematic of dosing schedule:**  Loading phase (Two days)  Maintenance phase (90 days – 7 day example) |
|  | 11b | Criteria for discontinuing or modifying allocated interventions for a given trial participant (eg, drug dose change in response to harms, participant request, or improving/worsening disease) Treatment Withdrawal: Participants will remain in the trial but should stop all trial treatments immediately upon the following:   - Diagnosis of COVID-19 on the basis of clinical symptoms and clinical judgement and/or on advice of treating clinician to stop taking the IMP. - Diagnosis of COVID-19 via validated NHS testing/other validated test  Participant Withdrawal Primary reasons for withdrawal may include:   - Suspected unexpected serious adverse reaction (SUSAR) or serious adverse event - Withdrawal of consent - participants may voluntarily withdraw from the study for any reason at any time - Lost to follow-up - Participants will be withdrawn at any time if the investigator concludes that it would be in the participant’s best interest for any reason. |
|  | 11c | Strategies to improve adherence to intervention protocols, and any procedures for monitoring adherence (eg, drug tablet return, laboratory tests)  Remote weekly review (phone, online-email, website or app based)  Electronic reminder alerts (e.g mobile phone texts) |
|  | 11d | Relevant concomitant care and interventions that are permitted or prohibited during the trial  See inclusion and exclusion criteria |
| Outcomes | 12 | Primary, secondary, and other outcomes, including the specific measurement variable (eg, systolic blood pressure), analysis metric (eg, change from baseline, final value, time to event), method of aggregation (eg, median, proportion), and time point for each outcome. Explanation of the clinical relevance of chosen efficacy and harm outcomes is strongly recommended  **Primary outcome:** Time to positive COVID-19 disease (based on positive symptoms)  **Secondary outcomes:** (per arm)  Number (and %) of COVID-19 test positive cases  Number of COVID-19 serological test positive cases  Severity of COVID-19 disease^δ^ (i) not requiring hospitalisation, requiring admission, HDU/ICU admission, death; (ii) length of personal sickness, length of inpatient stay [self-isolation due to contacts with positive household members will not be included] (iii) patient reported outcome measures^◊^  ^δ= based on medical records and participant feedback where appropriate^  ^◊ bameasures are taken weekly using telephone or online app/web-based interface for duration of participant is on the trial^  As this is a chemoprophylaxis trial, time to event (COVID-19 disease) is a suitable outcome measure to assess efficacy (or harm) of interventions (different hydroxychloroquine dosing regimens) compared to placebo.  The primary endpoint, time to disease onset, will be compared using a Cox proportional hazards model, adjusting with fixed effects for: age group (18-49, 50-59, 60+), sex, clinical area (high, medium and low COVID-19 exposure risk), site, known high-risk pre-existing conditions (e.g. cardiovascular disease, hypertension, diabetes, cancer, lung disease, immunosuppressed individuals), vs not. Estimates, 95% confidence intervals and p-values for the hazard ratios will be provided.  The primary comparison will be the placebo arm versus the pooled arms of hydroxychloroquine dosed daily and hydroxychloroquine dosed weekly. If, and only if, this primary comparison is statistically significant at a 5% significance level, will a further comparison will be made between the two active arms (hydroxychloroquine dosed daily versus hydroxychloroquine dosed weekly). Pooling the two hydroxychloroquine arms in a comparison with placebo will be sufficiently powered and is interpretable. |
| Participant timeline | 13 | Time schedule of enrolment, interventions (including any run-ins and washouts), assessments, and visits for participants. A schematic diagram is highly recommended (see Figure)  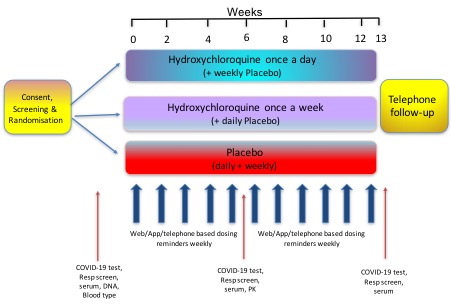 |
| Sample size | 14 | Estimated number of participants needed to achieve study objectives and how it was determined, including clinical and statistical assumptions supporting any sample size calculations  Assuming a hazard ratio of 0.7, and an infection rate of 0.3 at 3 months in the placebo arm, 261 events will provide 80% power under a 2-sided 5% significance level for the primary analysis. Assuming the recruitment period is very rapid and a conservative follow-up of 3 months, recruiting 250 placebo participants and 750 hydroxychloroquine participants (1000 in total), will provide the required number of events. The aim is to recruit 1200 participants to account for dropouts. |
| Recruitment | 15 | Strategies for achieving adequate participant enrolment to reach target sample size  Adverts, social media, trust wide information about the trial. |
| **Methods: Assignment of interventions (for controlled trials)** | | |
| Allocation: |  |  |
| Sequence generation | 16a | Method of generating the allocation sequence (eg, computer-generated random numbers), and list of any factors for stratification. To reduce predictability of a random sequence, details of any planned restriction (eg, blocking) should be provided in a separate document that is unavailable to those who enrol participants or assign interventions  Randomisation will be performed by a password secure online randomisation system. The system will provide a participant number and details of the investigational medicinal product (IMP) which will be used by the study team to prescribe the treatment allocated in a double-blinded manner. Randomisation will be in a 3:3:2 ratio hydroxychloroquine (daily), hydroxychloroquine (weekly), placebo), using stratified block randomisation. Random block sizes will be used, and stratification will be by study site. |
| Allocation concealment mechanism | 16b | Mechanism of implementing the allocation sequence (eg, central telephone; sequentially numbered, opaque, sealed envelopes), describing any steps to conceal the sequence until interventions are assigned  Interventions will assigned as described below (section 17a) and will avoid investigator and participant un-blinding. |
| Implementation | 16c | Who will generate the allocation sequence, who will enrol participants, and who will assign participants to interventions  Randomisation will be performed by a secure password protected website that will assign participants to interventions. |
| Blinding (masking) | 17a | Who will be blinded after assignment to interventions (eg, trial participants, care providers, outcome assessors, data analysts), and how  This is a double-blind trial. In order to maintain this blind, study medication will be labelled with a unique Medication Pack Code Number which will be assigned to the subject via the online randomisation system. It will not be possible for the study team to determine treatment allocation using laboratory data during the trial period.  All data (interim and full) analysis and statistical analysis will be performed blinded (masked) and completed as much as possible prior to unlocking of randomisation. |
|  | 17b | If blinded, circumstances under which unblinding is permissible, and procedure for revealing a participant’s allocated intervention during the trial  Emergency un-blinding: In the event of a valid medical or safety reason, the responsibility to break the treatment code resides solely with the treating clinician (i.e the Investigator or Sub-investigator). Investigators should note that the occurrence of an SAE should not routinely precipitate immediate unblinding.  The online secure randomisation system will be used for emergency un-blinding. Appropriately trained and delegated site staff will be given the necessary access rights and permission to access this facility. If un-blinding occurs, the trial medication (Hydroxychloroquine/Placebo) must be discontinued. |
| **Methods: Data collection, management, and analysis** | | |
| Data collection methods | 18a | Plans for assessment and collection of outcome, baseline, and other trial data, including any related processes to promote data quality (eg, duplicate measurements, training of assessors) and a description of study instruments (eg, questionnaires, laboratory tests) along with their reliability and validity, if known. Reference to where data collection forms can be found, if not in the protocol  Will be detailed in trial procedures manual (TPM) |
|  | 18b | Plans to promote participant retention and complete follow-up, including list of any outcome data to be collected for participants who discontinue or deviate from intervention protocols  Electronic reminders to facilitate participant retention and complete follow up. Contact details for trial team to enable participants to contact if any queries or concerns. |
| Data management | 19 | Plans for data entry, coding, security, and storage, including any related processes to promote data quality (eg, double data entry; range checks for data values). Reference to where details of data management procedures can be found, if not in the protocol  See protocol. A separate data management plan is proposed. |
| Statistical methods | 20a | Statistical methods for analysing primary and secondary outcomes. Reference to where other details of the statistical analysis plan can be found, if not in the protocol  See protocol. A separate statistical analysis plan is proposed. |
|  | 20b | Methods for any additional analyses (eg, subgroup and adjusted analyses)  See protocol. A separate statistical analysis plan is proposed. |
|  | 20c | Definition of analysis population relating to protocol non-adherence (eg, as randomised analysis), and any statistical methods to handle missing data (eg, multiple imputation)  See protocol. A separate statistical analysis plan is proposed. |
| **Methods: Monitoring** | | |
| Data monitoring | 21a | Composition of data monitoring committee (DMC); summary of its role and reporting structure; statement of whether it is independent from the sponsor and competing interests; and reference to where further details about its charter can be found, if not in the protocol. Alternatively, an explanation of why a DMC is not needed  See protocol |
|  | 21b | Description of any interim analyses and stopping guidelines, including who will have access to these interim results and make the final decision to terminate the trial  Interim analyses will be scheduled based on regular calendar time intervals, to give flexibility to adapt to external information. DMC meetings will occur regularly, with the ability to amend the frequency of meetings. The first interim analysis will be conducted at approximately 6 weeks after the first participant has been recruited, to assess recruitment and event rates. Interim analysis will also be recommended at a time shortly before recruitment is scheduled to be halted, to assess if the sample size assumptions are realistic.  The primary endpoint and primary comparison will be sequentially analysed at each interim analysis using a Lan-DeMets error-spending approach corresponding to symmetric 2-sided O’Brien-Fleming boundaries ([**https://doi.org/10.1002/sim.4780131308**](https://doi.org/10.1002/sim.4780131308)). The assumptions regarding the sample size (including the event rate and recruitment rate) may be reviewed and the total sample size and target number of events may be adapted. Conditional and Predictive power calculations may be used to inform any such sample size adaptations.  If the sequential interim analyses suggest a large positive, or negative, effect then the DMC may consider recommending early termination. |
| Harms | 22 | Plans for collecting, assessing, reporting, and managing solicited and spontaneously reported adverse events and other unintended effects of trial interventions or trial conduct  See protocol |
| Auditing | 23 | Frequency and procedures for auditing trial conduct, if any, and whether the process will be independent from investigators and the sponsor  Sponsor trial monitoring plan is proposed |
| Ethics and dissemination | | |
| Research ethics approval | 24 | Plans for seeking research ethics committee/institutional review board (REC/IRB) approval  REC 20/NW/0211   \|  \| \| --- \|   IRAS no: 281919 |
| Protocol amendments | 25 | Plans for communicating important protocol modifications (eg, changes to eligibility criteria, outcomes, analyses) to relevant parties (eg, investigators, REC/IRBs, trial participants, trial registries, journals, regulators)  See protocol  Before the start of the trial or implementation of any amendment we will obtain approval of the trial protocol, protocol amendments, informed consent forms and other relevant documents e.g., advertisements and GP information letters if applicable from the REC. All correspondence with the REC will be retained in the Trial Master File/Investigator Site File. Annual reports will be submitted to the REC in accordance with national requirements. |
| Consent or assent | 26a | Who will obtain informed consent or assent from potential trial participants or authorised surrogates, and how (see Item 32)  Trial investigators (participant information sheet and consent form will be provided). |
|  | 26b | Additional consent provisions for collection and use of participant data and biological specimens in ancillary studies, if applicable  See consent form |
| Confidentiality | 27 | How personal information about potential and enrolled participants will be collected, shared, and maintained in order to protect confidentiality before, during, and after the trial  All data will be transferred into a Case Report Form (CRF) which will be anonymised. All trial data in the CRF must be extracted from and be consistent with the relevant source documents. The CRFs must be completed, dated and signed by the investigator or designee in a timely manner. It remains the responsibility of the investigator for the timing, completeness, legibility and accuracy of the CRF pages. The CRF will be accessible to trial coordinators, data managers, the Investigators, Clinical Trial Monitors, Auditors and Inspectors as required.  A trial specific data management plan will describe in detail the data management processes using the CRF and the trial database.  All investigators and trial site staff involved in this trial must comply with the requirements of the Data Protection Act 2018 and Trust Policy with regards to the collection, storage, processing and disclosure of personal information and will uphold the Act’s core principles. |
| Declaration of interests | 28 | Financial and other competing interests for principal investigators for the overall trial and each study site  Nil |
| Access to data | 29 | Statement of who will have access to the final trial dataset, and disclosure of contractual agreements that limit such access for investigators  CCTU’s data analysts and statisticians |
| Ancillary and post-trial care | 30 | Provisions, if any, for ancillary and post-trial care, and for compensation to those who suffer harm from trial participation  Cambridge University Hospitals NHS Foundation Trust, as a member of the NHS Clinical Negligence Scheme for Trusts, will accept full financial liability for harm caused to participants in the clinical trial caused through the negligence of its employees and honorary contract holders. There are no specific arrangements for compensation should a participant be harmed through participation in the trial, but no-one has acted negligently. |
| Dissemination policy | 31a | Plans for investigators and sponsor to communicate trial results to participants, healthcare professionals, the public, and other relevant groups (eg, via publication, reporting in results databases, or other data sharing arrangements), including any publication restrictions  Ownership of the data arising from this trial resides with the Sponsor and their delegates. On completion of the trial the data will be tabulated and analysed and a final trial report prepared. |
|  | 31b | Authorship eligibility guidelines and any intended use of professional writers  No intention to use professional writers |
|  | 31c | Plans, if any, for granting public access to the full protocol, participant-level dataset, and statistical code |
| Appendices |  | Full protocol will be available. No plans for public access to participant level dataset or statistical code |
| Informed consent materials | 32 | Model consent form and other related documentation given to participants and authorised surrogates  See attached |
| Biological specimens | 33 | Plans for collection, laboratory evaluation, and storage of biological specimens for genetic or molecular analysis in the current trial and for future use in ancillary studies, if applicable  Biological samples that are collected in this trial will be securely stored for the duration of the trial and will be accessible to authorised trial staff only. During the trial samples will be analysed in a central laboratory. With permission from trial participants, any unused samples at the end of this trial will be stored for future tests related to this study, and for future approved research projects. |

*It is strongly recommended that this checklist be read in conjunction with the SPIRIT 2013 Explanation & Elaboration for important clarification on the items. Amendments to the protocol should be tracked and dated. The SPIRIT checklist is copyrighted by the SPIRIT Group under the Creative Commons “[Attribution-NonCommercial-NoDerivs 3.0 Unported](http://www.creativecommons.org/licenses/by-nc-nd/3.0/)” license.
